# Disease burden of varicella versus other vaccine-preventable diseases before introduction of vaccination into the national immunisation programme in the Netherlands

**DOI:** 10.2807/1560-7917.ES.2019.24.18.1800363

**Published:** 2019-05-02

**Authors:** Alies van Lier, Brechje de Gier, Scott A McDonald, Marie-Josée J. Mangen, Maarten van Wijhe, Elisabeth A.M. Sanders, Mirjam E. Kretzschmar, Hans van Vliet, Hester E. de Melker

**Affiliations:** 1 Centre for Infectious Disease Control, National Institute for Public Health and the Environment (RIVM), Bilthoven, Netherlands; 2 Department of Science and Environment, Roskilde University, Roskilde, Denmark; 3 Department of Pediatric Immunology and Infectious Diseases, Wilhelmina’s Children Hospital, University Medical Center Utrecht (UMCU), Utrecht, Netherlands; 4 Julius Center for Health Sciences and Primary Care, University Medical Center Utrecht (UMCU), Utrecht University, Utrecht, Netherlands

**Keywords:** varicella zoster virus infection, vaccine-preventable diseases, vaccines and immunisation, public health policy, disability-adjusted life years, DALY, prioritisation in public health

## Abstract

**Introduction:**

Estimating burden of disease (BoD) is an essential first step in the decision-making process on introducing new vaccines into national immunisation programmes (NIPs). For varicella, a common vaccine-preventable disease, BoD in the Netherlands was unknown.

**Aim:**

To assess national varicella BoD and compare it to BoD of other vaccine-preventable diseases before their introduction in the NIP.

**Methods:**

In this health estimates reporting study, BoD was expressed in disability-adjusted life years (DALYs) using methodology from the Burden of Communicable Diseases in Europe (BCoDE)-project. As no parameters/disease model for varicella (including herpes zoster) were available in the BCoDE toolkit, incidence, disease progression model and parameters were derived from seroprevalence, healthcare registries and published data. For most other diseases, BoD was estimated with existing BCoDE-parameters, adapted to the Netherlands if needed.

**Results:**

In 2017, the estimated BoD of varicella in the Netherlands was 1,800 (95% uncertainty interval (UI): 1,800–1,900) DALYs. Herpes zoster mainly contributed to this BoD (1,600 DALYs; 91%), which was generally lower than the BoD of most current NIP diseases in the year before their introduction into the NIP. However, BoD for varicella was higher than for rotavirus gastroenteritis (1,100; 95%UI: 440–2,200 DALYs) and meningococcal B disease (620; 95%UI: 490–770 DALYs), two other potential NIP candidates.

**Conclusions:**

When considering the introduction of a new vaccine in the NIP, BoD is usually estimated in isolation. The current approach assesses BoD in relation to other vaccine-preventable diseases’ BoD, which may help national advisory committees on immunisation and policymakers to set vaccination priorities.

## Introduction

Routine childhood vaccination programmes started in the beginning of the 20th century and after new vaccines became available, have been extended ever since. The Dutch national immunisation programme (NIP) was officially launched in 1957 with universal childhood vaccination against poliomyelitis, diphtheria, tetanus and pertussis. However, mass vaccination programmes had already begun in 1953 with vaccination against diphtheria, and from 1954 onwards this was combined with tetanus and pertussis. The NIP gradually expanded with vaccination against rubella (1974: girls only; 1987: all children), measles (1976), mumps (1987), *Haemophilus influenzae* type b (Hib) disease (1993), meningococcal C disease (2002), pneumococcal disease (2006), human papillomavirus (HPV) infection (2010: girls only), and hepatitis B (2011; before 2011 risk group vaccination was in place). Vaccination against meningococcal W disease (using meningococcal ACWY vaccine) has been implemented as an outbreak measure in 2018/19 pending further advice from the Health Council of the Netherlands.

In several countries, additional childhood vaccines against highly common diseases like varicella (e.g. United States, Germany) and rotavirus gastroenteritis (e.g. Belgium, Germany, United Kingdom (UK)) have been included in the NIP [1,2]. In the UK, vaccination against meningococcal B disease was recently also implemented [2].

In the decision-making process on introduction of a new vaccine into the NIP, the first criterion used by the Health Council of the Netherlands is the burden of disease (BoD) at population and at individual level. The subsequent criteria taken into account cover the effectiveness and safety of vaccination, acceptability of vaccination, efficiency of vaccination (including cost-effectiveness), and priority of vaccination. Each criterion is formulated on the assumption that the previous one is met [3].

Population BoD can be high if a disease is severe for affected individuals and/or affects a large number of people [3]. Although varicella has a mild disease course for the vast majority of cases, severe complications and mortality may occur [4]. Furthermore, nearly everyone in the Netherlands encounters the varicella zoster virus (VZV) during early life [5] and consequently is also at risk of virus reactivation later in life, resulting in herpes zoster (HZ).

To assess the potential value of adding varicella vaccination to the NIP, it is insightful to compare the BoD of varicella to the BoD of other vaccine-preventable diseases, before vaccination against the latter was introduced into the NIP. Therefore, the objectives of this study were (i) to estimate the current BoD of varicella; and (ii) to compare this to BoD estimates of various vaccine-preventable diseases before their inclusion in the NIP; keeping in mind however, that the timing of introduction into the NIP differs by disease. This study can serve as example in considerations to take for BoD when new vaccine candidates need to be assessed on eligibility for inclusion in a NIP.

## Methods

In this health estimates reporting study, the BoD of the following vaccine-preventable diseases was estimated: diphtheria, pertussis, tetanus, poliomyelitis, measles, mumps, rubella, Hib disease, meningococcal C/W disease, pneumococcal disease, cervical cancer (HPV-infection), hepatitis B (current NIP diseases), meningococcal B disease, rotavirus gastroenteritis, and varicella (potential NIP candidates). The BoD of these diseases was estimated for the year prior to their introduction into the NIP, or for the year 2017 for potential NIP candidates, mainly using the Burden of Communicable Diseases in Europe (BCoDE) toolkit version 0.94 [6] and the parameters presented in Supplement 1. Additionally, when the incidence/BoD of a given disease was estimated to be higher in any of the five preceding years (when data were available), the BoD for the year with the highest incidence/BoD was also presented as an alternative, higher estimate of the BoD.

### Disability-adjusted life years methodology

BoD was expressed in disability-adjusted life years (DALYs) [7] (or DALYs per 100,000 population). This composite measure combines morbidity (years lived with disability; YLD) and mortality (years of life lost; YLL) in a single measure of health loss, allowing comparison between diseases with varying severity and incidence (e.g. rare with high mortality vs common with short self-recovery). The underlying methodology, outcome trees, and clinical progression probabilities have been described elsewhere [8-11].

The BCoDE toolkit does not include rotavirus gastroenteritis, varicella and cervical cancer.

For rotavirus gastroenteritis, a model developed by Havelaar et al. specific for the Dutch situation was used [11].

For varicella, a new disease progression model was developed. Dutch data on VZV seroprevalence [12], general practitioner (GP) consultations [13], hospitalisations [14] and mortality [15] were used to determine the proportion of mild (no contact with healthcare)/moderate (GP consultation)/severe (hospitalisation) varicella, and the mortality risk. Congenital varicella syndrome was not included as Dutch VZV seroprevalence at childbearing age is close to 100% [5], and the syndrome only occurs in 0.4–2.0% of all children born to mothers with varicella during the first 20 weeks of gestation [4]. The BoD of varicella and HZ are often investigated separately; however, because these two diseases are so closely related, the BoD of HZ was also estimated. For HZ the disease model previously developed by Kristensen et al. [16] (using disability weights from Salomon et al. [17] and Kwong et al. [18]) for people aged ≥ 50 years was used. It was extended with parameters for people aged < 50 years and the Global Burden of Disease (GBD) 2010 life expectancy (LE) [19] was applied (Supplement 1, Tables A4.13/A1).

For cervical cancer the BoD estimates of McDonald et al. were used [20]. Note that the estimates derived using this method are not fully comparable to the other diseases: a different life table (Dutch LE 2014) and source for disability weights (Victorian BoD study) [21] were employed and BoD was computed from the number of registered cervical cancer cases and deaths and the HPV-attributable fraction, instead of deriving BoD from the number of incident HPV infections (which is unknown).

Except for cervical cancer and HZ, the European disability weights elicited by Haagsma et al. [22], and the GBD 2010 LE [19] were applied, in contrast to previous BoD estimates [9-11].

To test the validity of the standard case fatality parameters, historical mortality data were obtained. For diphtheria and tetanus, the original model estimated significantly fewer deaths than registered, whereas it estimated significantly more deaths than registered for poliomyelitis and measles. Therefore, for diphtheria, poliomyelitis and measles, YLL was estimated based on registered instead of estimated mortality, assuming one additional future measles death due to subacute sclerosing panencephalitis (SSPE). For tetanus, we considered it likely that each reported case in 1953 (and 1950) – before availability of mechanical ventilation/intensive care – died of the disease [23] rather than using mortality data, which may be unreliable for tetanus in that period. Furthermore, the percentage of the paralytic form of poliomyelitis was based on notification data rather than literature-derived estimates (Supplement 1, Tables A4).

### Incidence data

Incidence data were derived from various sources and adjusted for underestimation using multiplication factors (Supplement 1, Table A2/A3). The incidence of varicella was estimated based on transmission modelling of Dutch VZV seroprevalence data [12], the incidence of HZ was estimated based on incidence data of the Netherlands Institute for Health Services Research (NIVEL) [13]. For some diseases only cases with *invasive* disease caused by serotypes covered by the vaccine were included, i.e.**Hib, meningococcus C/W/B and pneumococcus 1, 4, 5, 6B, 7F, 9V, 14, 18C, 19F, and 23F (PCV10 serotypes). For cervical cancer BoD was scaled by 71% because vaccine serotypes HPV-16/18 are estimated to be responsible for 71% of invasive cervical cancer [24]. For hepatitis B, only BoD attributable to new infections in 2010 was estimated; BoD due to infections acquired before the year 2010 was excluded because the BoD associated with such infections can no longer be prevented by vaccination.

Cases with unknown age and/or sex were imputed using the univariate method. Prior to introduction of vaccination into the NIP, complete information was not always available on the distribution of incidence by sex and 5-year age group. For example, if the highest age group was ‘20 years or older’ these cases were assigned to the age group ‘20–24 years’ because for most diseases incidence was highest among young people in the pre-vaccination period. If sex was unknown, cases were divided equally between males and females (Supplement 1, Table A2).

### Sensitivity analysis and uncertainty

In the baseline analysis the GBD 2010 LE was used. However, in the 1950s LE was ca 10 years lower than nowadays. To allow a comparison of health loss due to a disease across time, the BoD was also estimated with the year-specific Dutch LE [25] for the total population (no distinction men/women; Supplement 1, Table A1), except for cervical cancer for which the Dutch LE 2014 was used [20].

Statistical uncertainty (e.g. due to small sample size) was simulated using Monte Carlo techniques (5,000 iterations were run per disease model) and results are presented as the mean and 95% uncertainty intervals (UI) resulting from the stochastic simulations (see van Lier et al. [10]). DALY estimates were rounded to three significant digits for numbers ≥ 10,000, to two significant digits for numbers between 10 and 10,000 and to one significant digit for numbers < 10.

### Ethical statement

All data used in this study were aggregated and non-identifiable; therefore, ethical approval was not required. 

## Results

**Figure 1:**
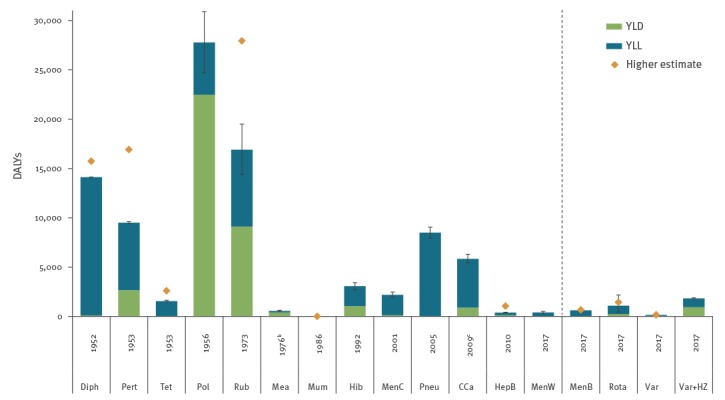
Estimated disease burden^a^ of vaccine-preventable diseases in the year before introduction of vaccination into the national immunisation programme, or in 2017, with the years lived with disability and the years of life lost components shown separately, Netherlands, 1952–2017

The estimated BoD of various vaccine-preventable diseases in the Netherlands in the year before introduction of vaccination into the NIP, or in 2017, are shown in Figure 1. BoD for varicella alone was estimated at 160 (95%UI: 160–160 (rounded)) DALYs but amounted to 1,800 (95%UI: 1,800–1,900) DALYs including HZ. This was higher than the BoD for rotavirus gastroenteritis (1,100; 95%UI: 440–2,200 DALYs) and meningococcal B disease (620; 95%UI: 490–770 DALYs), the two other potential NIP candidates.

CCa: cervical cancer (human papillomavirus (HPV)-16/18); DALY: disability-adjusted life year; Diph: diphtheria; HepB: hepatitis B;  Hib: invasive *Haemophilus influenzae* type b disease;  HZ: herpes zoster; Mea: measles;  MenC/W/B: invasive meningococcal C/W/B disease; Mum: mumps;  NIP: national immunisation programme; Pert: pertussis; Pneu: invasive pneumococcal disease (PCV10 types); Pol: poliomyelitis;  Rota: rotavirus gastroenteritis; Rub: rubella; Tet: tetanus; Var: varicella; YLD: years lived with disability; YLL:  years of life lost.

^a^ Disease burden is expressed in DALYs.

^b^ 1976 because there were no 1975 data.

^c^ Dutch life expectancy in 2014 instead of global burden of disease (GBD) 2010 life expectancy.

Left of vertical dashed line: estimates for the year before inclusion in the NIP; right of vertical dashed line: estimates for 2017 for potential NIP candidates. Whiskers indicate 95% uncertainty intervals for DALYs. A higher estimate (orange dot) is given if the burden in DALYs in any of the 5 years preceding the year before inclusion in the NIP is higher than that year. In this case this higher estimate is the highest burden in DALYs in the five preceding years; see manuscript and Supplement 1 for all assumptions and limitations.

In the year before introduction into the NIP, the estimated BoD was highest for poliomyelitis (27,800; 95%UI: 24,700–30,900 DALYs), rubella (16,900; 95%UI: 14,400–19,500 DALYs) and diphtheria (14,100; 95%UI: 14,100–14,100 (rounded) DALYs), followed by pertussis (9,500; 95%UI: 9,400–9,600 DALYs), invasive pneumococcal disease (8,500; 95%UI: 8,000–9,100 DALYs) and cervical cancer (5,800; 95%UI: 5,400–6,300 DALYs), and relatively low for mumps (4; 95%UI: 3–4 DALYs). Figure 1 illustrates that for most diseases, the BoD in the year before introduction of vaccination was highest or very similar to the highest BoD in any of the five preceding years, except from rubella and pertussis where BoD estimates in the year with the highest incidence were 27,900 (95%UI: 24,000–32,200) and 16,900 (95%UI: 16,700–17,100) DALYs respectively.

Figure B1 (Supplement 2) shows that BoD per 100,000 population for diseases for which vaccination was introduced in the 1950s was relatively high compared with absolute BoD, due to the smaller population size in that period.

**Figure 2:**
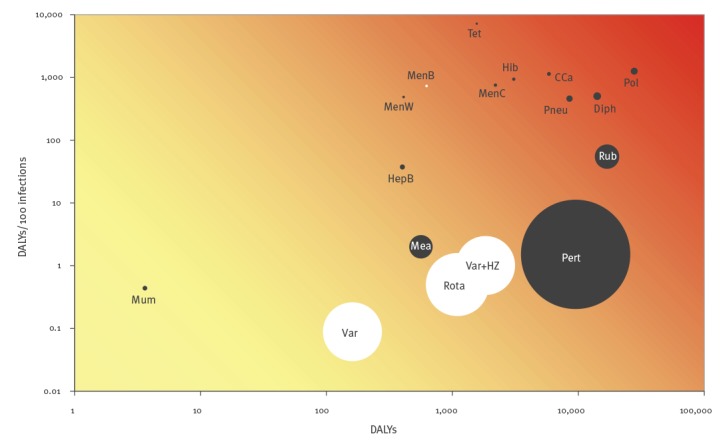
Ranking of vaccine-preventable diseases by estimated disease burden^a^ at population and individual level in the year before introduction of vaccination into the national immunisation programme, or in 2017, Netherlands, 1952–2017

**Figure 3:**
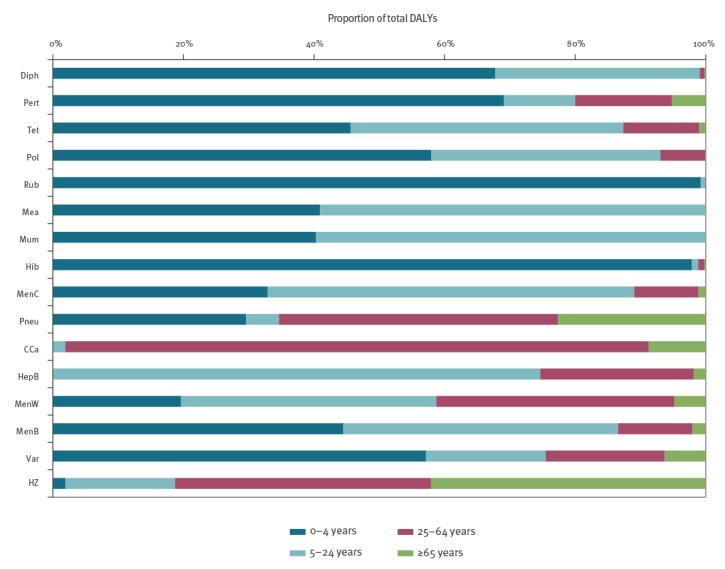
Estimated proportion of disease burden^a^ of vaccine-preventable diseases by age group in the year before introduction of vaccination into the national immunisation programme, or in 2017, Netherlands, 1952–2017

Most vaccine-preventable diseases included in the current NIP (Figure 2, black bubbles) had a relatively high BoD at both the population and the individual level in the year before introduction of vaccination into the NIP. The potential NIP candidates (Figure 2, white bubbles) rotavirus gastroenteritis and varicella (including HZ) had a relatively low BoD at individual level in 2017 but a considerable number of cases were affected, whereas meningococcal B/W disease and hepatitis B had a relatively high BoD at the individual level with a limited number of cases. Figure 3 shows that, with the exception of invasive pneumococcal disease, cervical cancer and HZ, the BoD of vaccine-preventable diseases mainly occurred among the youngest age groups (< 25 years).

CCa: cervical cancer (human papillomavirus (HPV)-16/18); DALY: disability-adjusted life year; Diph: diphtheria; HepB: hepatitis B; Hib: invasive *Haemophilus influenzae* type b disease; HZ: herpes zoster; Mea: measles; MenC/W/B: invasive meningococcal C/W/B disease; Mum: mumps; NIP: national immunisation programme; Pert: pertussis; Pneu: invasive pneumococcal disease (PCV10 types); Pol: poliomyelitis; Rota: rotavirus gastroenteritis; Rub: rubella; Tet: tetanus; Var: varicella.

^a^ Disease burden is expressed in DALYs.

Both axes are on a logarithmic scale. Black bubbles represent estimates for the year before inclusion in the NIP. White bubbles represent estimates for 2017 for potential NIP candidates. The area of each bubble is proportional to the average number of estimated cases (250 cases were added to each bubble for visibility reasons). The gradient colouring from the lower left quadrant to the upper right quadrant is used to indicate different levels of burden of disease (yellow: relatively low burden at population and individual level, i.e. mumps; red: relatively high burden at population and individual level, i.e. poliomyelitis); see manuscript and Supplement 1 for all assumptions and limitations.

CCa: cervical cancer (human papillomavirus (HPV)-16/18); DALY: disability-adjusted life year; Diph: diphtheria; HepB: hepatitis B; Hib: invasive *Haemophilus influenzae* type b disease; HZ: herpes zoster; Mea: measles; MenC/W/B: invasive meningococcal C/W/B disease; Mum: mumps; Pert: pertussis; Pneu: invasive pneumococcal disease (PCV10 types); Pol: poliomyelitis; Rub: rubella; Tet: tetanus; Var: varicella.

^a^ Disease burden is expressed in DALYs.

Rotavirus gastroenteritis could not be included in this figure as generation of age-specific DALY estimates was not foreseen in the model of Havelaar et al. [11]. Zooming in on the BoD for varicella and HZ per 100,000 population, it is evident that the highest BoD for varicella was estimated among young children < 5 years old and for HZ among people aged ≥ 50 years (Supplement 2, Figure B3).

### Sensitivity analysis

Although the estimates incorporating year-specific Dutch LE for the total population in place of GBD 2010 LE were somewhat lower, the ranking of diseases in terms of BoD before introduction of vaccination into the NIP was identical (Supplement 2, Figure B2).

## Discussion

To our knowledge, this is the first attempt to estimate the BoD of various vaccine-preventable diseases before introduction of vaccination into the NIP using DALYs. This adds to work of Van Wijhe et al. who analysed the impact of vaccination programmes on mortality burden and notified cases [26,27], and work of Colzani et al. on the impact of vaccination on measles BoD [28].

Notwithstanding the possibility of severe complications, varicella is generally perceived as having a mild course (4.1 on a scale from 1 = not severe at all to 10 = very severe [29]). Various surveillance data – GP consultations, hospitalisations and mortality – reflect the severity of varicella [13,30,31], which contributes to the overall burden. However, as diseases and their consequences are heterogeneous, it is difficult to compare the overall burden of varicella with the overall burden of other vaccine-preventable diseases. We attempted to address this by using the composite health measure DALY. The current BoD of varicella in the Netherlands expressed in DALYs was relatively low compared with the BoD of most other vaccine-preventable diseases in the year before their introduction into the NIP. However, BoD of varicella and HZ combined was higher than for rotavirus gastroenteritis and meningococcal B disease, two other potential candidates for inclusion in the NIP.

Survey data showed that only a minority of both parents and professionals in the Netherlands favour universal varicella vaccination through the NIP [32-34]. In the most recent study, only 21% of professionals had a positive attitude towards universal varicella vaccination, and 28% of parents had a positive intention to vaccinate their own child against varicella if included within the NIP [34]. Justification for these opinions is that varicella was perceived as being neither important nor severe enough for vaccination to be needed [29,34]. The current BoD estimates may challenge these notions of professionals and parents on the usefulness of varicella vaccination.

Parents’ intentions were more positive towards universal vaccination against rotavirus gastroenteritis (38% positive) and especially meningococcal B disease (83% positive) [29]. According to Veldwijk et al. potential coverage for rotavirus vaccination would range between 23 and 86%, depending on vaccine scenario (vaccine effectiveness, protection duration) and implementation strategy [35].

While childhood vaccination against varicella may result in a reduction of varicella BoD, this might increase HZ BoD in the mid-term because it has been hypothesised that reduced VZV circulation reduces exogenous immune boosting, thereby increasing the probability of HZ [12]. On the other hand, vaccination against varicella will possibly diminish HZ among vaccinated individuals because the vaccine-strain is less likely to reactivate than the wild-type strain [36]. Therefore, cost-effectiveness of varicella vaccination in the Netherlands depends strongly on the impact on HZ and the economic time horizon. In the absence of exogenous immune boosting, vaccination with high coverage is expected to be cost-effective and may even be cost saving, while it is not expected to be cost-effective on reasonable time scales (< 100 years) if immune boosting is present [12].

Alternatively, VZV BoD could be reduced by vaccination in middle-age against HZ [37] which could be marginally cost-effective in the Netherlands, depending on the vaccine price [38-40]. HZ vaccination would likely not affect the high VZV circulation among young children and thus maintain the benefits of early infection: less severe disease following primary infection and preventing susceptibility among women of reproductive age.

Diseases situated in the upper right quadrant of Figure 2 – most vaccine-preventable diseases included in the current NIP – have a relatively high BoD at both population and individual level, justifying inclusion in the NIP. Due to the limited number of cases, the Figure might generate more discussion regarding vaccination against hepatitis B and meningococcal W/B disease, but as the individual-level burden of these diseases is high, vaccination can still be relevant. With the availability of an affordable combination vaccine, hepatitis B vaccination was included in the NIP because there was more health gain with universal vaccination compared with the former risk group vaccination, at low additional costs [41]. Vaccination against meningococcal W disease was mainly introduced as an outbreak measure because of the sharp increasing incidence and high case fatality rate [42]. Diseases in the lower right quadrant (high BoD at population level but low BoD at the individual level) raise more discussion (most potential NIP diseases). Diseases in the lower left quadrant would only end up in the NIP when additional costs are very low or when vaccination is cost-saving. Although the BoD of mumps was modest at the time mumps vaccination was added to the NIP, it was included in the MMR vaccine through which vaccination against measles, rubella, and mumps could easily be combined [43]. Rabies (very severe and rare) is an example of a vaccine-preventable disease that would appear in the upper left quadrant.

BoD is the first criterion used by the Health Council of the Netherlands to determine a vaccine’s suitability for inclusion in the NIP. Other criteria cover effectiveness and safety of vaccination, acceptability of vaccination, efficiency of vaccination (including cost-effectiveness), and priority of vaccination [3]. In 2007, the council advised to further review the inclusion of vaccination against varicella, rotavirus gastroenteritis and meningococcal B disease once more information became available [44]. The current study provides valuable information on the BoD of these diseases. Recently, the council recommended vaccination against rotavirus gastroenteritis, while noting that universal vaccination is not cost-effective at current vaccine prices whereas risk-group vaccination is considered to be cost-saving [45,46].

The principal strength of this study is the utilisation of extensive historical data on the incidence and mortality of vaccine-preventable diseases. Furthermore, we applied a standardised BoD methodology using publicly available software and sets of outcome trees.

Interpretation of our findings should recognise several limitations.

First, the year of introduction of vaccination into the NIP differed across diseases; consequently it is not straightforward to compare the situation in the 1950s with the situation of today: healthcare and treatment options, immunisation status of the population and surveillance (notification criteria, laboratory testing) have changed significantly over this period, as has the population demographics. For example, the risk of dying from poliomyelitis or measles is very low nowadays and the proportion of elderly has increased. Regardless of the year of estimation, the same outcome tree was used, as relevant data were not available to adjust the clinical progression probabilities over time (with the exception of adjustments to case-fatality rates when the estimated number of deaths was considerably different from the registered number of deaths). As a consequence, BoD was probably underestimated for diseases for which vaccination was introduced many years ago. At the same time, BoD for these diseases was likely overestimated through use of the same LE (baseline) for all diseases, regardless of the year in which vaccination was introduced. Although LE at birth increased by approximately 10 years since the 1950s [25,47], we considered it unjust to value a life in the 1950s differently from a life today. Despite this concern, the sensitivity analysis showed that using year-specific Dutch LE did not have a large impact on the results.

A second limitation is that BoD was estimated for a single year, even though incidence can fluctuate over time (e.g. outbreaks) (Supplement 1, Figures A1.1–A1.17). An extreme example is diphtheria for which there were tens of thousands of cases annually during the Second World War period. There were fewer, reported cases however, in 1952 (n = 2,805), the year used in this study [48]. For poliomyelitis it is the other way around: BoD was estimated from 2,206 reported cases in 1956, whereas in 1957 only 203 cases were reported [48]. However, we showed that for most diseases, the BoD in the year before introduction of vaccination was highest or very similar to the highest BoD in any of the five preceding years, except from rubella and pertussis.

A third limitation is that only *invasive* Hib, meningococcal and pneumococcal disease were included in this estimation. Although vaccination was primarily introduced to prevent invasive disease, our estimates only cover a limited part of the total BoD (excluding for example pneumonia and otitis media) caused by these pathogens. A similar observation can be made for HPV: this vaccination is expected to also prevent cancers other than cervical cancer. In addition, cross-protection effects against serotypes not covered by the HPV vaccine [49] were not included.

Finally, the BoD might have been underestimated because of different reasons or assumptions. The BoD of diphtheria, tetanus and pertussis might be underestimated because vaccination already started before introduction of mass vaccination in 1953 [50]. Pertussis BoD was probably also underestimated due to under-reporting of deaths [51] (used to estimate incidence), and the assumed age distribution of cases (outbreak 2012) which was probably not comparable to the year 1953 (i.e. higher age of infection in 2012 than in 1953 as a result of the NIP). The BoD of invasive Hib disease is expected to be underestimated as Bol estimated more cases than were reported [52]. BoD of measles and rubella might be underestimated as well by using reported cases (corrected for underestimation based on outbreaks in 1999–2000 and 2013–14) while almost every child contracted these diseases in the pre-vaccination period [48]. Varicella BoD might be slightly overestimated because we based the incidence on seroprevalence data while some cases might be asymptomatic. However, underestimation of varicella BoD because of not including long-term sequelae due to congenital varicella syndrome is more likely.

Taken together, the BoD results presented in this manuscript must be seen as rough estimates: the exact value of these estimates is less relevant than the ratio of diseases to each other. The latter is less likely to change.

In conclusion, the present-day BoD of varicella – including HZ – in the Netherlands is somewhat lower than the BoD of most vaccine-preventable diseases before their inclusion in the NIP, but higher than the BoD of other potential NIP candidates. Based on established BoD estimation methods, the current approach provides a quantitative evidence base for decision-making regarding the inclusion of new vaccines – such as varicella vaccine – in NIPs. Whereas the introduction of a new vaccine into NIPs is usually assessed in isolation, the current analysis provides insight into the BoD of different diseases in relation to each other, which can be helpful for national advisory committees on immunisation (NITAGs) and policymakers to prioritise vaccination programmes.

## Supplementary Data

919000 Supplement 1

## Supplementary Data

404000 Supplement 2
