# Supplementary material for: Disease burden of varicella versus other vaccine-preventable diseases before introduction of vaccination into the national immunisation programme in the Netherlands
Source: Euro Surveill. 2019 May 2;24(18):1800363. doi: 10.2807/1560-7917.ES.2019.24.18.1800363 (PMC6505181; doi:10.2807/1560-7917.ES.2019.24.18.1800363)
Supplement: Supplement 1 [file 1800363_VanLIER_Supplement1.pdf]

This supplementary material is hosted by *Eurosurveillance* as supporting information alongside the article 'Disease burden of varicella versus other vaccine-preventable diseases before introduction of vaccination into the national immunisation programme in the Netherlands' by van Lier et al. on behalf of the authors who remain responsible for the accuracy and appropriateness of the content. The same standards for ethics, copyright, attributions and permissions as for the article apply. *Eurosurveillance* is not responsible for the maintenance of any links or email addresses provided therein.

Supplement 1 Disease burden model parameters

Table A1 Life expectancies used in baseline analysis (GBD 2010 life expectancy[a] [1]) and sensitivity analysis (Dutch life expectancy[b] for specific years [2], interpolated at average ages of death)

Age-group	GBD	Netherlands										
(years)	2010	1952	1953	1956	1973	1976	1986	1992	2001	2005	2010	2017[c]
0	85.68	72.22	71.78	72.60	74.21	74.70	76.37	77.35	78.32	79.47	80.80	81.52
1–4	83.63	71.97	71.49	72.05	73.02	73.46	74.92	75.79	76.68	77.81	79.05	79.74
5–9	78.76	67.44	67.00	67.44	68.31	68.72	70.14	70.99	71.85	72.97	74.20	74.88
10–14	73.79	62.59	62.20	62.59	63.43	63.82	65.20	66.04	66.89	68.00	69.23	69.91
15–19	68.83	57.73	57.39	57.70	58.56	58.93	60.27	61.11	61.96	63.05	64.27	64.95
20–24	63.88	52.91	52.59	52.87	53.79	54.12	55.41	56.24	57.08	58.15	59.35	60.02
25–29	58.94	48.12	47.82	48.07	48.98	49.30	50.57	51.39	52.20	53.25	54.44	55.11
30–34	54.00	43.34	43.05	43.26	44.16	44.46	45.72	46.54	47.34	48.36	49.53	50.21
35–39	49.09	38.61	38.34	38.49	39.37	39.66	40.89	41.73	42.49	43.51	44.65	45.34
40–44	44.23	33.93	33.68	33.80	34.66	34.91	36.14	36.97	37.71	38.70	39.83	40.48
45–49	39.43	29.36	29.13	29.20	30.09	30.31	31.49	32.31	33.04	33.99	35.07	35.71
50–54	34.72	24.98	24.76	24.78	25.68	25.90	27.01	27.80	28.51	29.43	30.46	31.04
55–59	30.10	20.80	20.58	20.54	21.53	21.69	22.76	23.45	24.14	25.01	26.01	26.55
60–64	25.55	16.85	16.66	16.62	17.59	17.76	18.79	19.38	19.97	20.78	21.75	22.24
65–69	21.12	13.24	13.05	13.03	14.02	14.20	15.11	15.64	16.05	16.79	17.72	18.16
70–74	16.78	10.04	9.88	9.78	10.85	11.04	11.82	12.26	12.54	13.11	13.93	14.32
75–79	12.85	7.40	7.26	7.13	8.11	8.30	8.98	9.32	9.46	9.88	10.53	10.80
80–84	9.34	5.35	5.24	5.04	5.87	6.06	6.57	6.83	6.84	7.16	7.63	7.71
85+	5.05	3.24	3.15	3.04	3.42	3.59	3.87	3.97	3.91	4.05	4.25	4.21

[a] For the Global Burden of Disease (GBD) 2010 study, it was decided to use the same reference standard for males and females and to use a life table based on the lowest observed death rate for each age group in countries of more than 5 million in population, [b] the total Dutch life expectancy was taken following the GBD 2010 study which did not distinguish between men and women, [c] based on Dutch life expectancy in 2016 because life expectancy for 2017 was not yet available.

Table A2 Reported[a] number of new cases before introduction of vaccination in the national immunisation programme (NIP) or in 2017

Disease	Year in NIP	Year burden estimation [b]	Reported cases [a,b]	Data sources	Assumptions on distribution by sex/age
Diphtheria	1953	1952 (1950)	2,805 (2,985)	IGZ [3]	50% male, 50% female; 50+=50–54 years of age.
Pertussis	1954	1953 (1950)	17,386 (31,122)	CBS [4]	As pertussis has only been notifiable since 1975, incidence was back calculated from the disease model using the 81 deaths registered in 1953 (145 in 1950). Distribution of the estimated reported cases by sex/age was based on notifications from the 2012 outbreak, distribution of deaths by sex/age on registered deaths in 1953 (1950).
Tetanus	1954	1953 (1952)	18 (32)	IGZ [5]	50% male, 50% female; 30–39=divided in 50% 30–34 + 50% 35–39 years of age; 40–49=divided in 50% 40–44 + 50% 45–49 years of age; 60–79=divided in 25% 60–64 + 25% 65–69 years of age + 25% 70–74 + 25% 75–79 years of age.
Poliomyelitis	1957	1956 (n.a.)	2,206	IGZ [6]	50% male, 50% female; 30–39=divided in 50% 30–34 + 50% 35–39 years of age; 40–49=divided in 50% 40–44 + 50% 45–49 years of age; 50–59=divided in 50% 50–54 + 50% 55–59 years of age; 70–79=divided in 50% 70–74 + 50% 75–79 years of age.
Rubella	1974	1973 (1968)	2,715 (4,935)	IGZ/OSIRIS [4, 7]	50% male, 50% female; 10–19=divided in 10–14 + 15–19 years of age based on age distribution in 1975; 20–29=divided in 50% 20–24 + 50% 25–29 years of age; 30+=30–34 years of age; cases with missing age imputed.
Measles	1976	1976 (n.a.)	[c] 2,512	IGZ [8]	50% male, 50% female; 20+=20–24 years of age; cases with missing age imputed.
Mumps	1987	1986 (1983)	458 (822)	OSIRIS [4]	50% male, 50% female; 00–04=divided in 0 + 1–4 years of age and 10–19=divided in 10–14 + 15–19 years of age based on age distribution in 1978, 20+=20–24 years of age; cases with missing age imputed.
I. *Haemophilus influenzae* type b disease	1993	1992 (n.a.)	291	Osiris/NRLBM	
I. meningococcal C disease	2002	2001 (n.a.)	277	Osiris/NRLBM	
I. pneumococcal disease (PCV10 types)	2006	2005 (n.a.)	[d] 1,648	Osiris/NRLBM	
Cervical cancer	2010	2009 (n.a.)	[e] 723	NKR/CBS	
Hepatitis B infection (acute)	2011	2010 (2005)	199 (301)	Osiris	
Invasive meningococcal W disease	2018	2017 (n.a.)	80	Osiris/NRLBM	
Invasive meningococcal B disease	[f]	2017 (2013)	81 (88)	Osiris/NRLBM	
Rotavirus gastroenteritis	[f]	2017 (2013)	[g] 2,755 (3,937)	Sentinel laboratory surveillance	50% male, 50% female; incidence estimates for infectious gastroenteritis (GE) are based on population-based cohort studies in the nineteen-nineties as described in Havelaar et al. [9-11]
Varicella /	[f]	2017 (2012)	182x1,000 (185x1,000)	Seroprevalence VZV [12]	Estimation based on transmission modelling of Dutch varicella-zoster virus (VZV) seroprevalence data, applied to Dutch population in 2017 (2012) [13].
Herpes zoster		2017 (n.a.)	89x1,000	NIVEL [14]	Estimation based on incidence data 2015, applied to Dutch population in 2017.

[a] Pertussis, cervical cancer, rotavirus gastroenteritis, varicella and herpes zoster were not notifiable and therefore alternative data sources were used to estimate the incidence, [b] in parentheses the year and number of cases of an alternative estimate when the incidence and burden in DALYs was higher in one of the five preceding years before the original estimate, [c] data for the year 1976 (1975 not available), [d] corrected for 25% coverage of the surveillance system, [e] 71% of the burden of these cases was included in the disease burden estimates, [f] potential candidates for inclusion into the NIP, [g] 16.1 per 100,000 inhabitants in 2017 (23.3 per 100,000 in 2013) [15].

CBS=Statistics Netherlands, IGZ=the Health Care Inspectorate, NIVEL=Netherlands Institute for Health Services Research, NKR=Netherlands Cancer Registry, NRLBM=Netherlands Reference laboratory for Bacterial Meningitis, Osiris=Dutch surveillance system for notifiable infectious diseases.

Figure A1.1 Number of reported cases of diphtheria 1950–2017

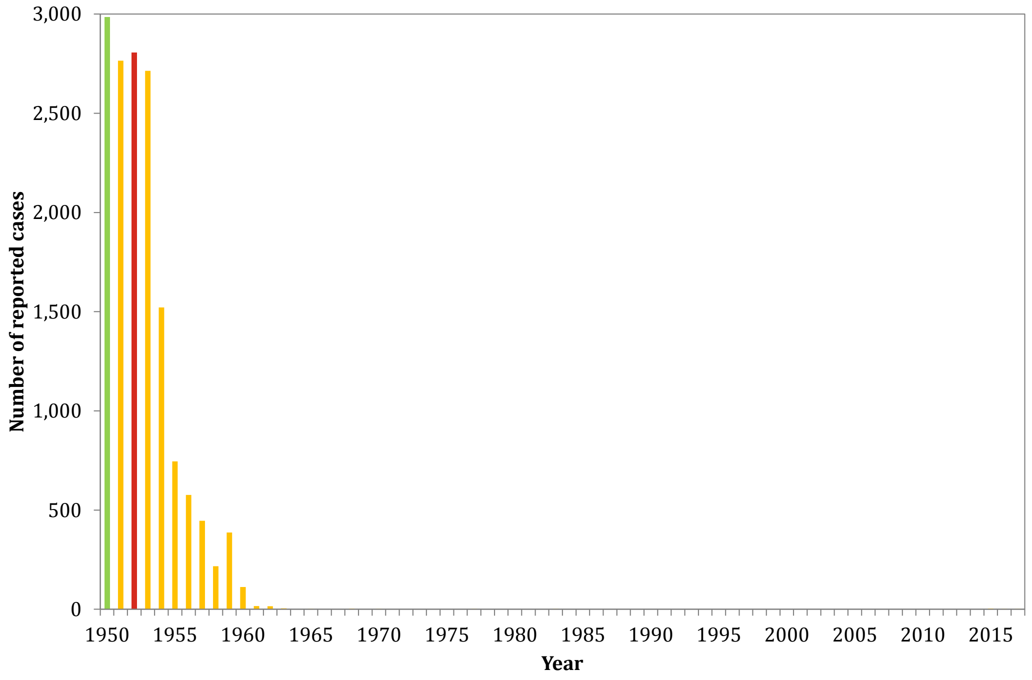

The red bar indicates the year of disease burden calculation (the green bar the year of the alternative higher burden estimate).
Source: https://www.volksgezondheidenzorg.info/onderwerp/ziekten-het-rijksvaccinatieprogramma/cijfers-context/overzicht

Figure A1.2 Number of reported cases of pertussis 1976–2017

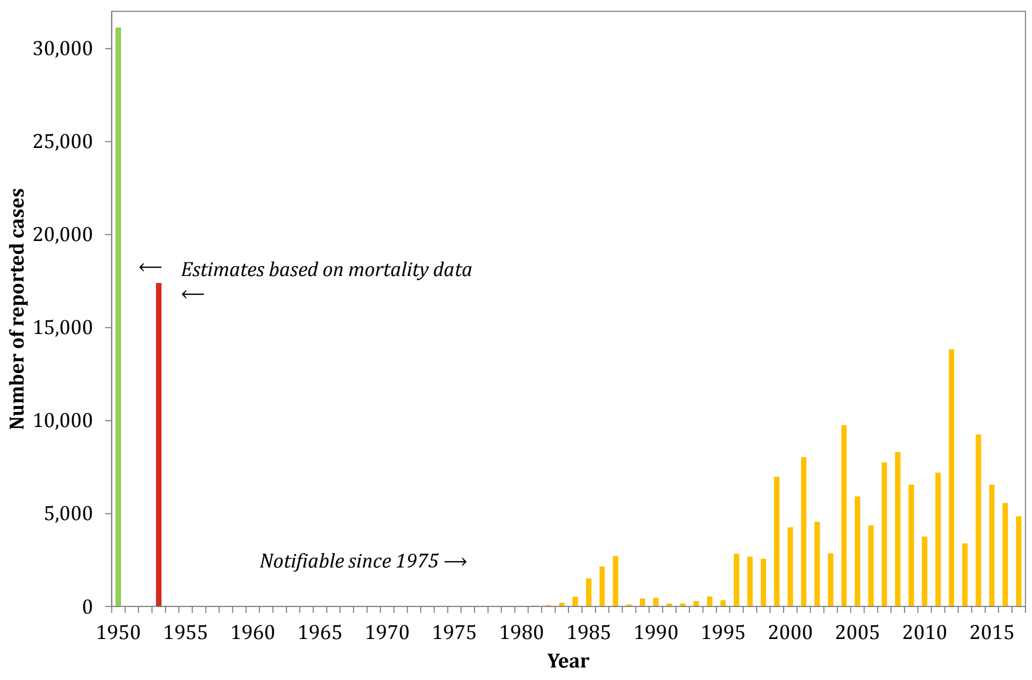

The red bar indicates the year of disease burden calculation (the green bar the year of the alternative higher burden estimate).
As pertussis has only been notifiable since 1975, the incidence in 1953 (and 1950) was backcalculated from mortality data.
Source: https://www.volksgezondheidenzorg.info/onderwerp/ziekten-het-rijksvaccinatieprogramma/cijfers-context/overzicht

Figure A1.3 Number of reported cases of tetanus 1952–2017

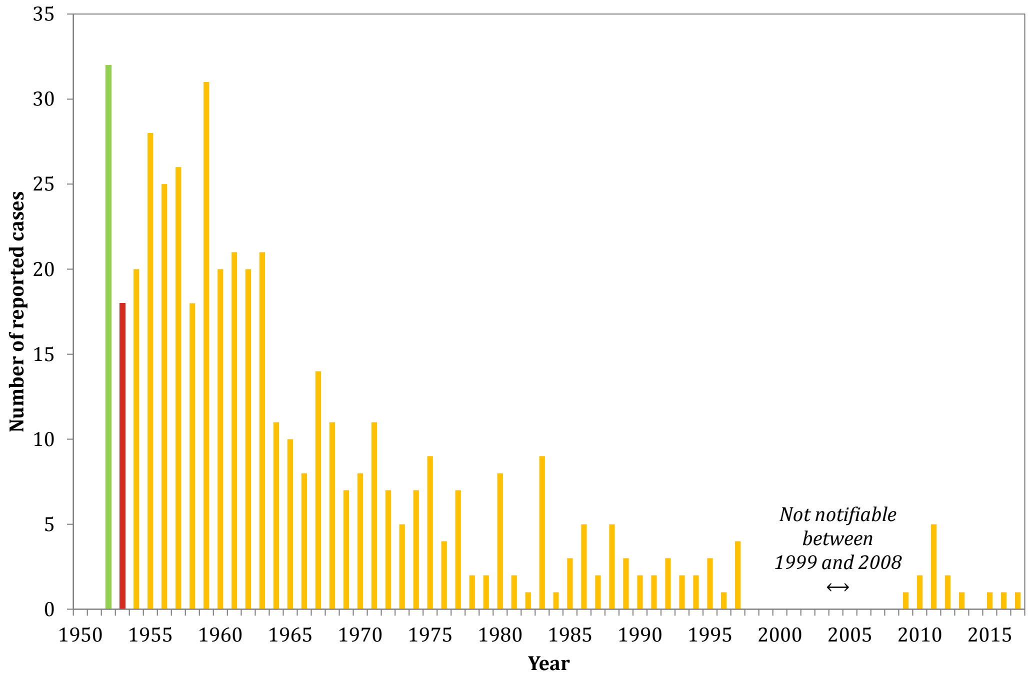

The red bar indicates the year of disease burden calculation (the green bar the year of the alternative higher burden estimate).
Source: https://www.volksgezondheidenzorg.info/onderwerp/ziekten-het-rijksvaccinatieprogramma/cijfers-context/overzicht

Figure A1.4 Number of reported cases of poliomyelitis 1950–2017

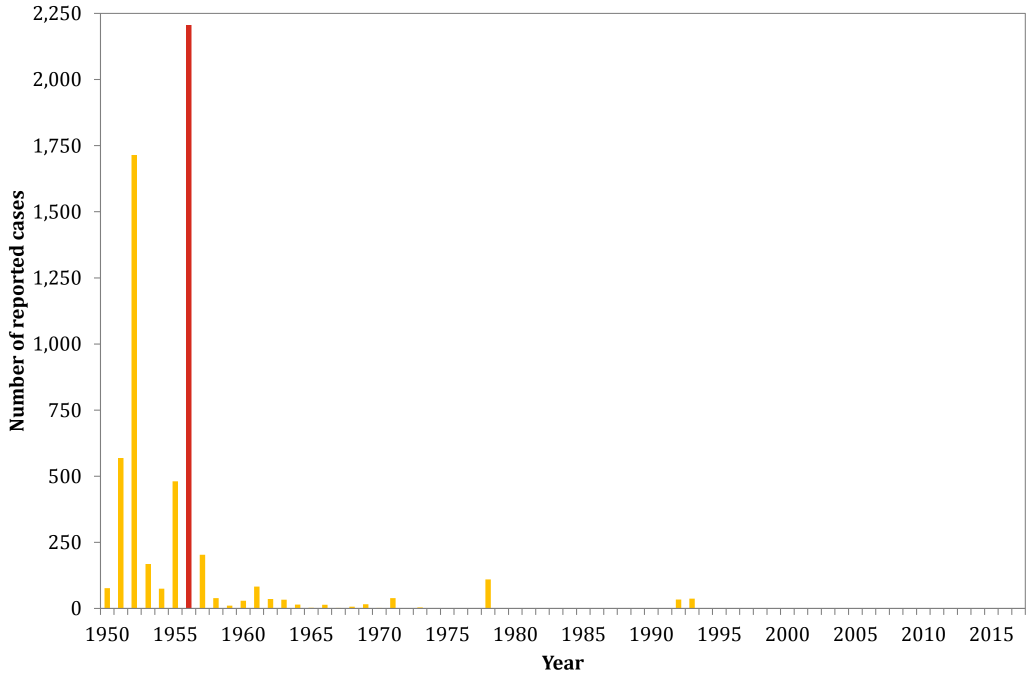

The red bar indicates the year of disease burden calculation.
Source: https://www.volksgezondheidenzorg.info/onderwerp/ziekten-het-rijksvaccinatieprogramma/cijfers-context/overzicht

Figure A1.5 Number of reported cases of rubella 1952–2017

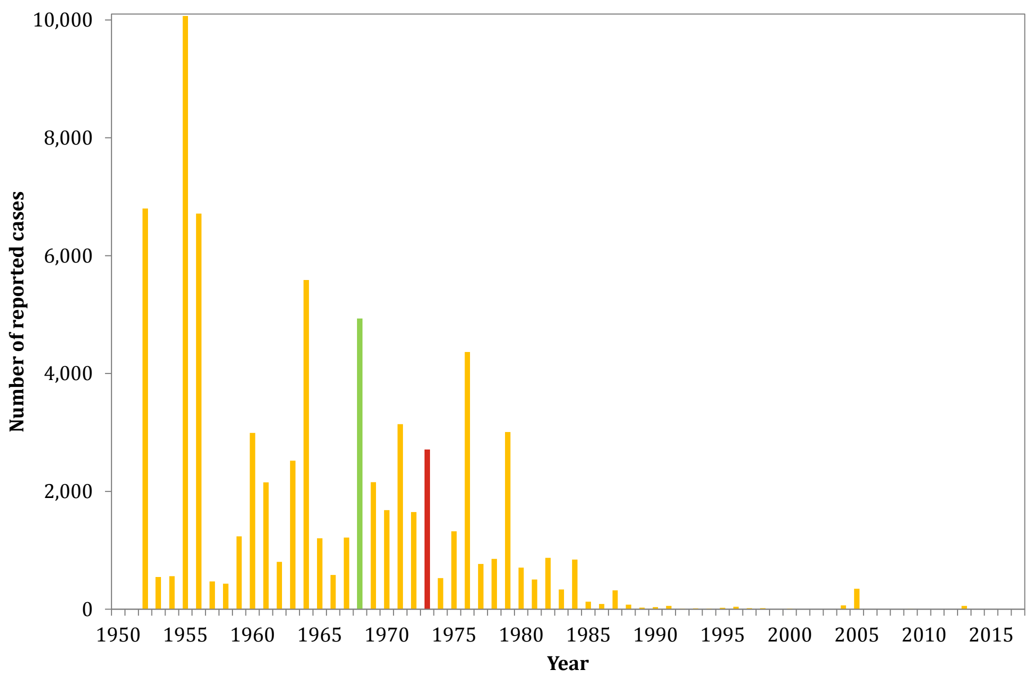

The red bar indicates the year of disease burden calculation (the green bar the year of the alternative higher burden estimate).
Sources: https://www.volksgezondheidenzorg.info/onderwerp/ziekten-het-rijksvaccinatieprogramma/cijfers-context/overzicht
Van den Hof S, Conyn-van Spaendonck MAE, de Melker HE, Geubbels ELPE, Suijkerbuijk AWM, Talsma E, et al. The effects of vaccination, the incidence of the target diseases. Bilthoven: National Institute for Public Health and the Environment (RIVM); 1998 (RIVM report 213676008).

Figure A1.6 Number of reported cases of measles 1976–2017

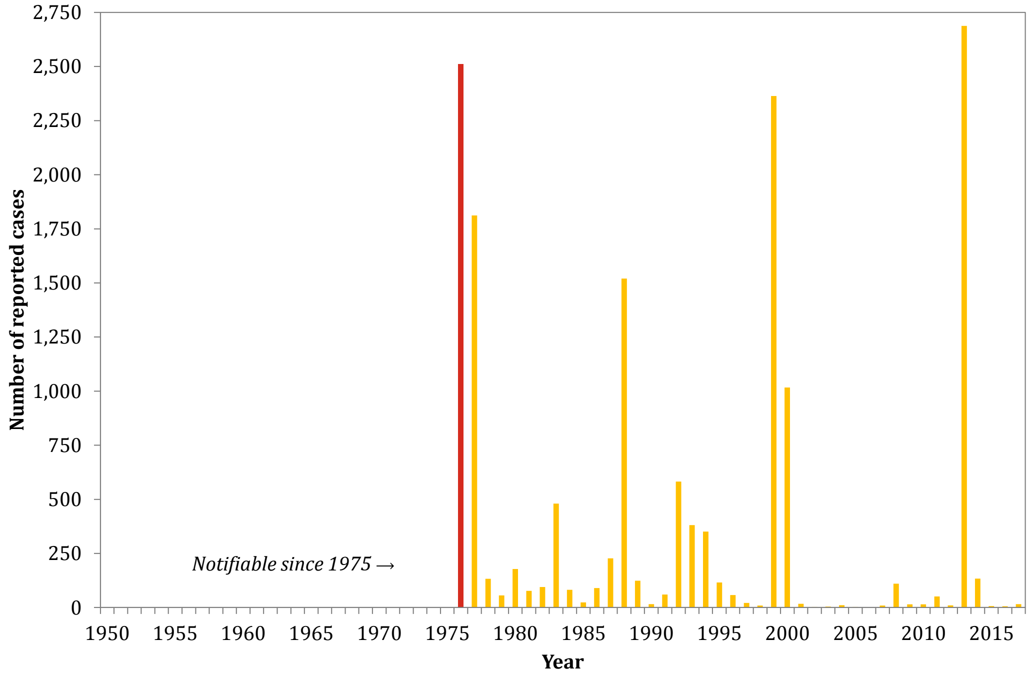

The red bar indicates the year of disease burden calculation.
Source: https://www.volksgezondheidenzorg.info/onderwerp/ziekten-het-rijksvaccinatieprogramma/cijfers-context/overzicht

Figure A1.7 Number of reported cases of mumps 1976–2017

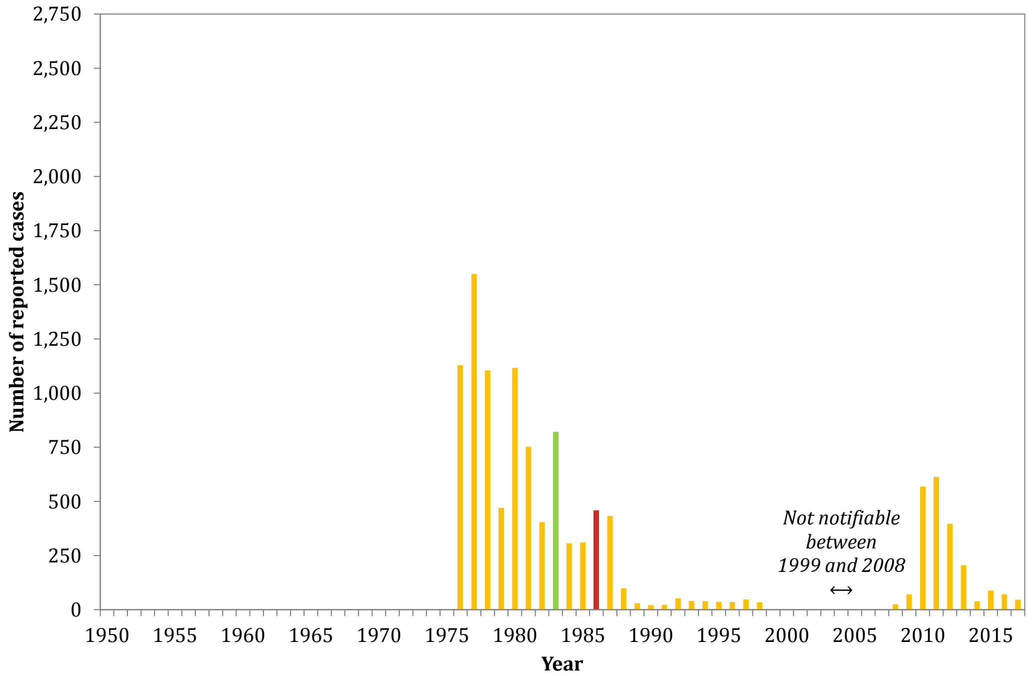

The red bar indicates the year of disease burden calculation (the green bar the year of the alternative higher burden estimate).
Source: https://www.volksgezondheidenzorg.info/onderwerp/ziekten-het-rijksvaccinatieprogramma/cijfers-context/overzicht

Figure A1.8 Number of reported cases of invasive *Haemophilus influenzae* type b disease 1992–2017

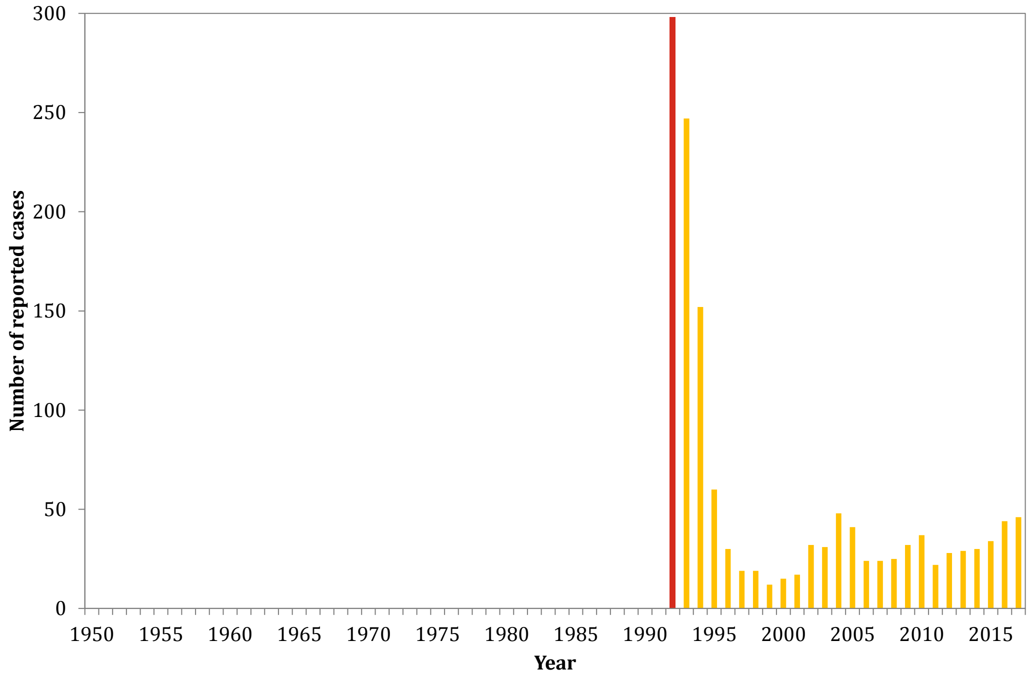

The red bar indicates the year of disease burden calculation.
Source: https://www.volksgezondheidenzorg.info/onderwerp/ziekten-het-rijksvaccinatieprogramma/cijfers-context/overzicht

Figure A1.9 Number of reported cases of invasive meningococcal C disease 1992–2017

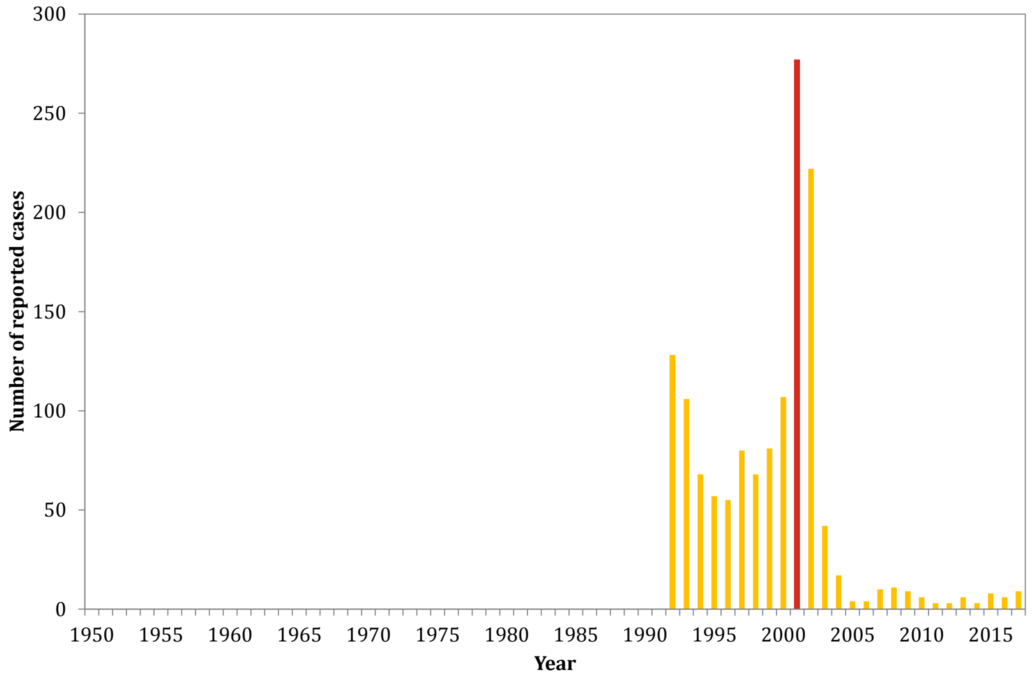

The red bar indicates the year of disease burden calculation.
Source: https://www.volksgezondheidenzorg.info/onderwerp/ziekten-het-rijksvaccinatieprogramma/cijfers-context/overzicht

Figure A1.10 Number of reported cases of invasive pneumococcal disease (PCV10 types) 2004–2017

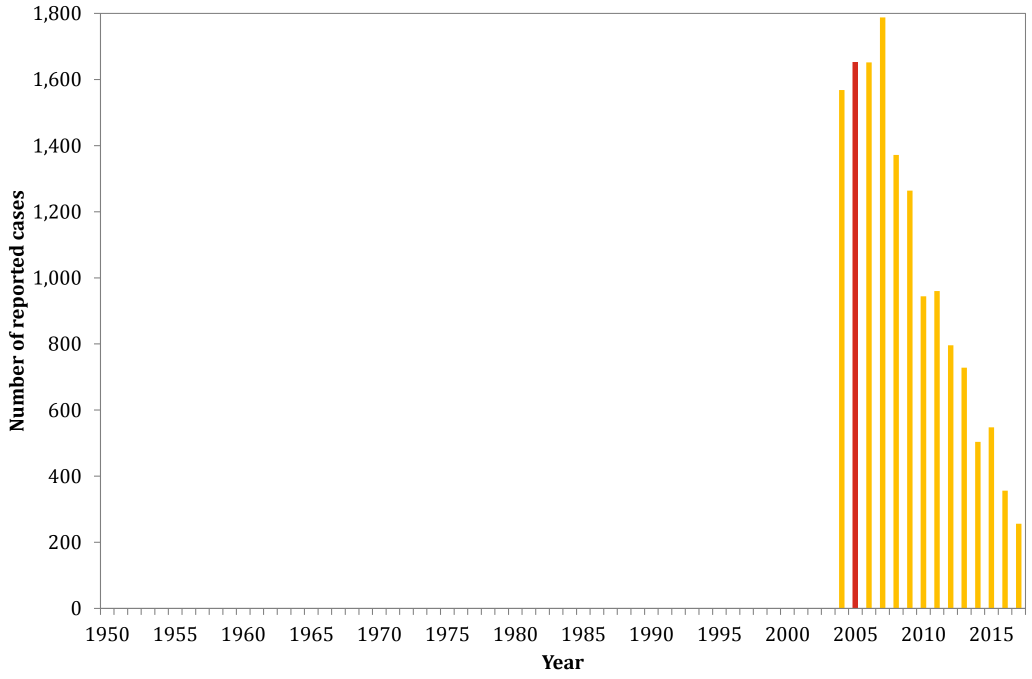

The red bar indicates the year of disease burden calculation.
Source: OSIRIS/NRLBM

Figure A1.11 Number of reported new cases of cervical cancer 1989–2017

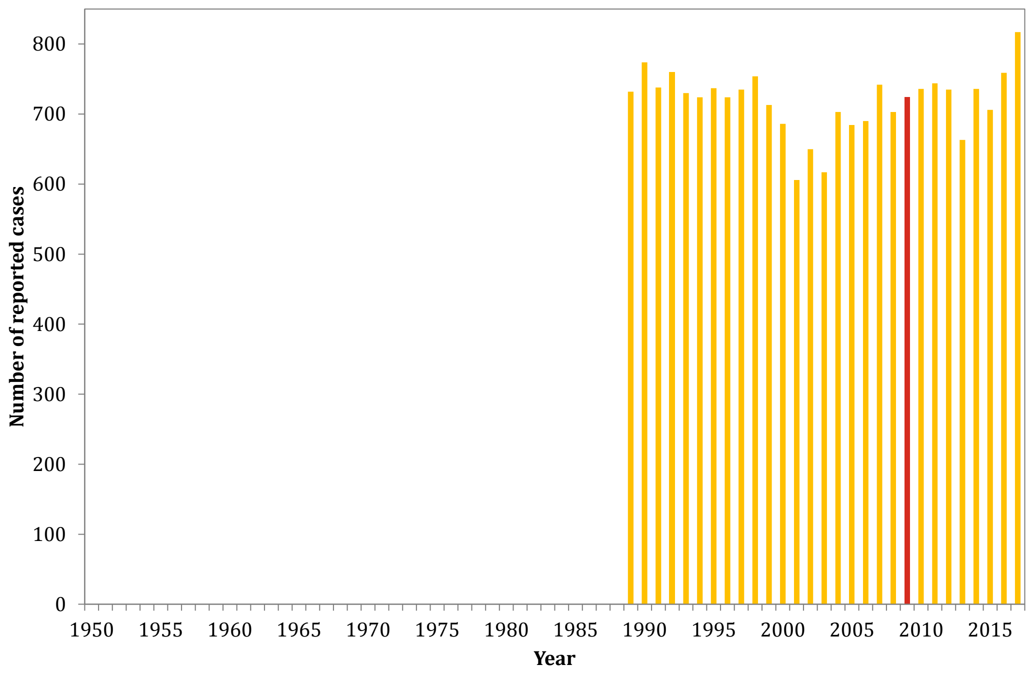

The red bar indicates the year of disease burden calculation (no alternative higher burden estimate because the burden in DALYs in 2007 is somewhat lower than in 2009, despite the slightly higher number of cases due to difference in mortality figures between these years).
Source: https://www.cijfersoverkanker.nl (2017; preliminary data)

Figure A1.12 Number of reported cases of acute hepatitis B infection 1976–2017

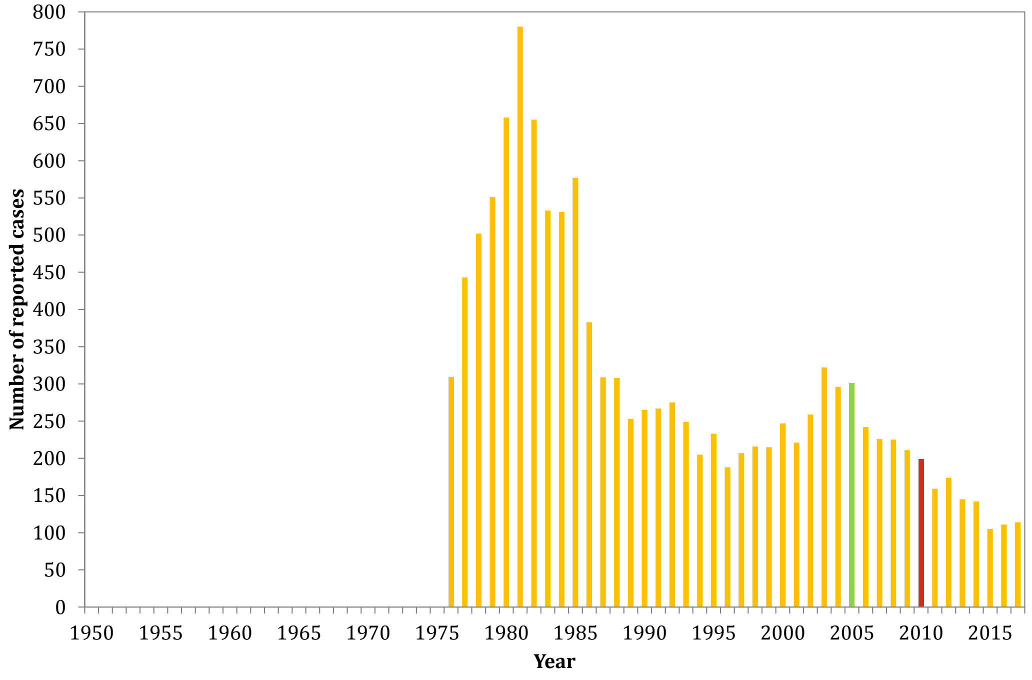

The red bar indicates the year of disease burden calculation (the green bar the year of the alternative higher burden estimate).
Source: IGZ/OSIRIS

Figure A1.13 Number of reported cases of invasive meningococcal W disease 1992–2017

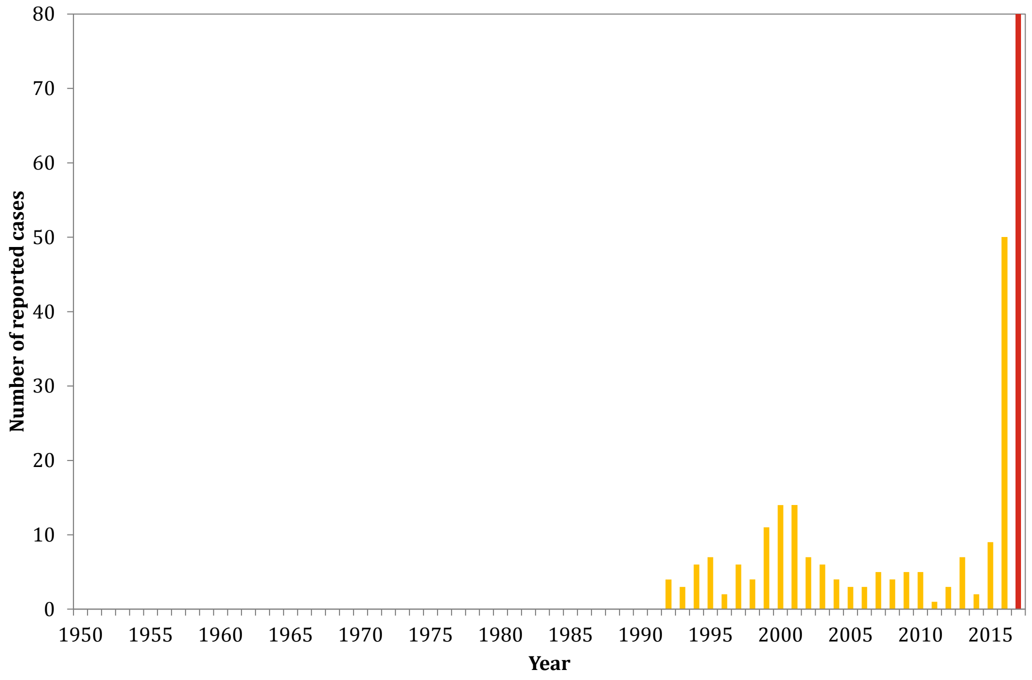

The red bar indicates the year of disease burden calculation.
Source: OSIRIS/NRLBM

Figure A1.14 Number of reported cases of invasive meningococcal B disease 1992–2017

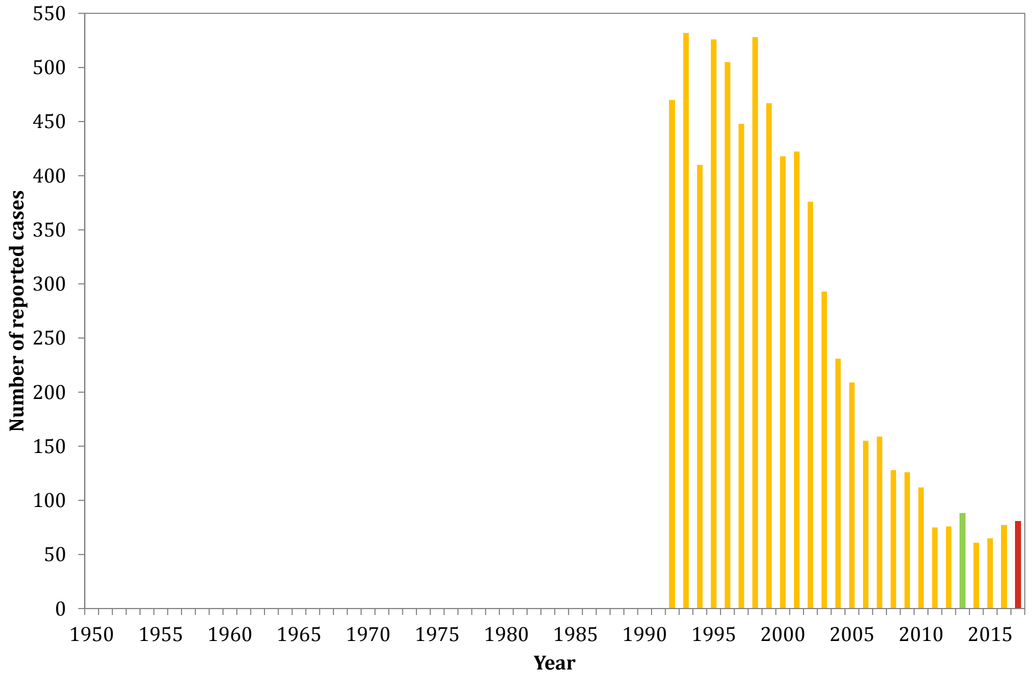

The red bar indicates the year of disease burden calculation (the green bar the year of the alternative higher burden estimate).
Source: OSIRIS/NRLBM

Figure A1.15 Incidence per 100,000 of rotavirus gastroenteritis 1999–2017

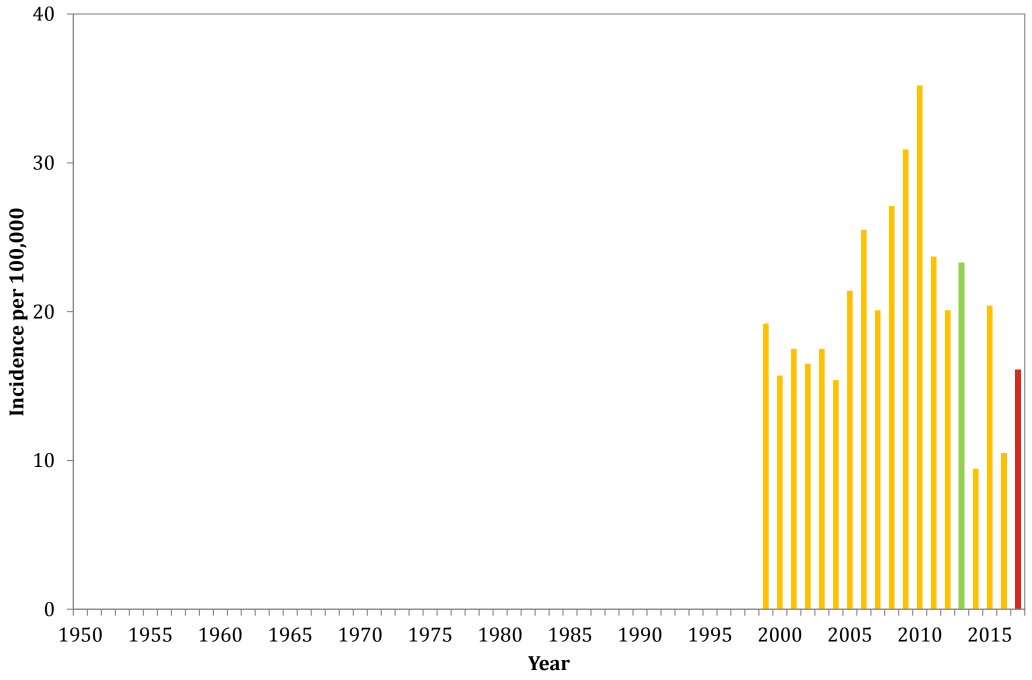

The red bar indicates the year of disease burden calculation (the green bar the year of the alternative higher burden estimate).
Source: Mangen MJ, Friesema IHM, Pijnacker R, Mughini Gras L, van Pelt W. Disease burden of food-related pathogens in the Netherlands, 2017. Bilthoven: RIVM; 2018 (RIVM Letter report 2018-0037). http://www.rivm.nl/bibliotheek/rapporten/2018-0037.pdf

Figure A1.16 Estimated number of cases of varicella 2000–2017

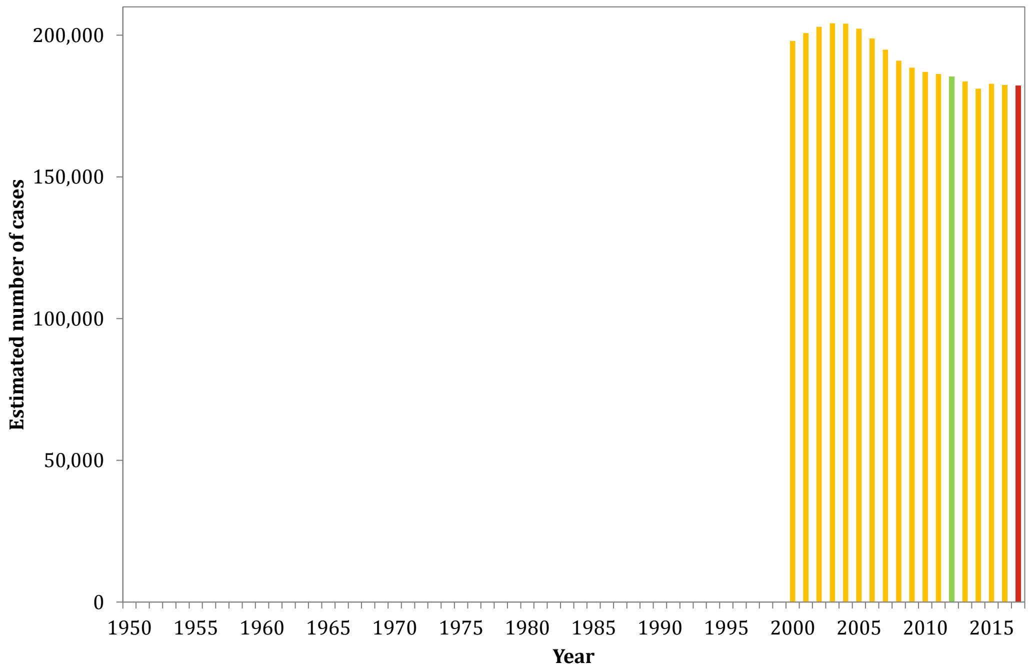

The red bar indicates the year of disease burden calculation (the green bar the year of the alternative higher burden estimate).
Source: van Lier A, Lugnér A, Opstelten W, Jochemsen P, Wallinga J, Schellevis F, et al. Distribution of Health Effects and Cost-effectiveness of Varicella Vaccination are Shaped by the Impact on Herpes Zoster. EBioMedicine. 2015;2(10):1494-9.

Figure A1.17 Estimated number of cases of herpes zoster 2000–2017

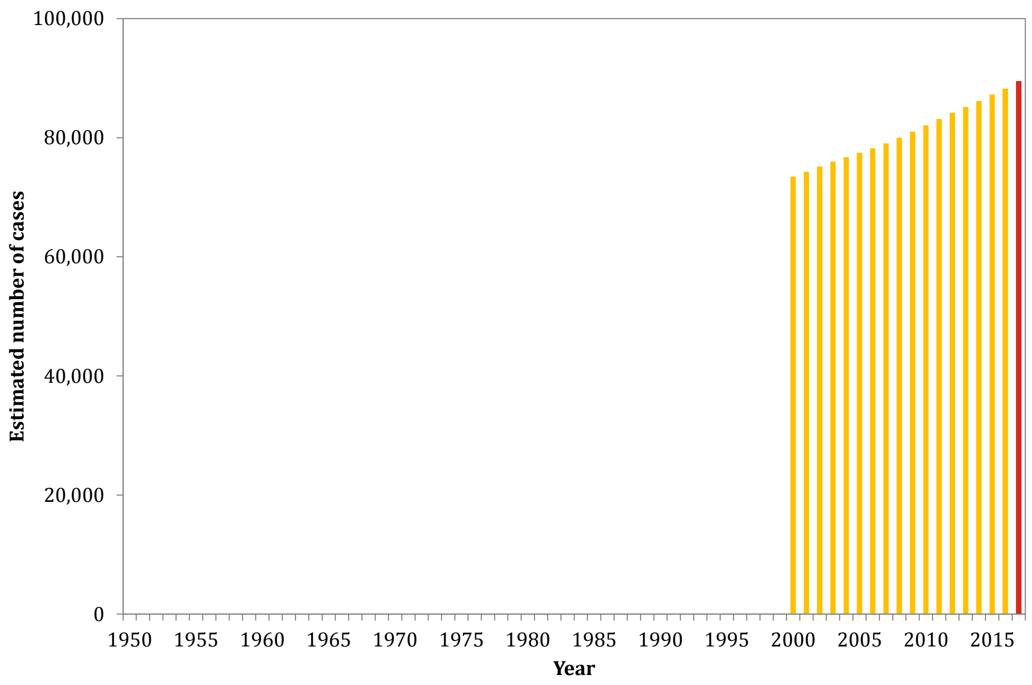

The red bar indicates the year of disease burden calculation.
Source: Estimation based on NIVEL incidence data 2015, applied to Dutch population 2000–2017

Table A3 Multiplications factors to adjust for under-estimation[a]

Disease	Multiplication factor (MF)	Source/assumption
Diphtheria	UE: 1	No MF available.
Hepatitis B infection (acute)	UA: 1.33<br>UR: Uniform(1.20,1.22)	For UE, we assume that 75% of all symptomatic cases are reported; this is based on England and Wales data from 1992-1996 [16]. For UR, the lower bound was derived from a 1996 audit, and the upper bound was taken from Swaan et al.'s (unpub.) study of reporting completeness (weighted mean of 83.1%) by 13 Zuid-Holland laboratories, 2005-2010.
Invasive *Haemophilus influenzae* disease	UE: Uniform(1.05,1.20)	Approximately 83-95% of isolates are sent to the Netherlands Reference Laboratory for Bacterial Meningitis and this leads to an MF of 1.05 to 1.20 (expert opinion).
Invasive meningococcal disease (C/W/B)	UE: 1.05	Meningococcal disease is a notifiable disease and notifications are cross-checked with data of the Netherlands Reference Laboratory for Bacterial Meningitis. Therefore coverage will be very high, around 95%. This leads to an MF of 1.05 (expert opinion).
Invasive pneumococcal disease	UE: Uniform(1.05,1.20)	Approximately 83-95% of isolates are sent to the Netherlands Reference Laboratory for Bacterial Meningitis and this leads to an MF of 1.05 to 1.20 (expert opinion).
Measles	UE: Pert(8.44,11.21,15.02)	MF based on random effects meta-analysis of data from measles outbreaks in 1999/2000 [17] and 2013/2014 (preliminary data).
Mumps	UE: Pert(1.55,1.79,2.13)	MF based on random effects meta-analysis of data from mumps outbreaks in 2009/2010 [18] and 2012 [19].
Pertussis	UE: Pert(23,41,66) (<1 yr)<br>Pert(17,25,34) (1-4 yrs)<br>Pert(16,26,39) (5-9 yrs)<br>Pert(6,10,15) (10-19 yrs)<br>Pert(37,47,59) (20-59 yrs)<br>Pert(49,69,96) (60+ yrs)	MF derived by evidence synthesis approach [20].
Poliomyelitis	UE: 1	Because of the severity of the disease, we assumed that all cases are identified. We assumed no disease burden for asymptomatic cases.
Rotavirus infection	UE (estimated):<br>77.4 (95%UI: 40.3–134.8)	For these foodborne diseases, an estimation method developed by Havelaar et al. was used that is specific for the Dutch situation [9-11]. UE was estimated based on difference between number of estimated cases (N=137,618) and number of reported cases (N=1,779).
Rubella	UE: Pert(8.44,11.21,15.02)	MF of measles used as proxy (the clinical picture is probably less clear compared with measles, except for congenital rubella syndrome).
Tetanus	UE: Uniform(1.0,1.41)	Range of 1 to 1.41 was based on expert opinion that the MF would be close to 1.0 (set as lower bound), and a Danish study suggesting 1.41 (upper bound) [21].
Varicella /	UE: 1	No MF applied: number of cases based on transmission modelling of Dutch varicella-zoster virus (VZV) seroprevalence data [13].
Herpes zoster	UE: Pert(0.87,0.91,0.94)	The majority of patients will consult their GP because it is a painful condition. The positive predictive value of clinical judgement is 90.8% (95%CI: 87.3%–94.3%) [22].

Uniform=minimum and maximum value, Pert=minimum, most likely and maximum value.

[a] Under-estimation (UE) can be divided in under-ascertainment (UA, refers to the extent to which incidence is under-estimated because there are cases in the community that do not get in contact with health services, such as their general practitioner) and under-reporting (UR, refers to those infected individuals who do contact health services, but whose disease status is incorrectly diagnosed or classified, or fails to be reported to the organisation responsible for surveillance).

Table A4.1 Outcome tree and disease specific parameters: diphtheria

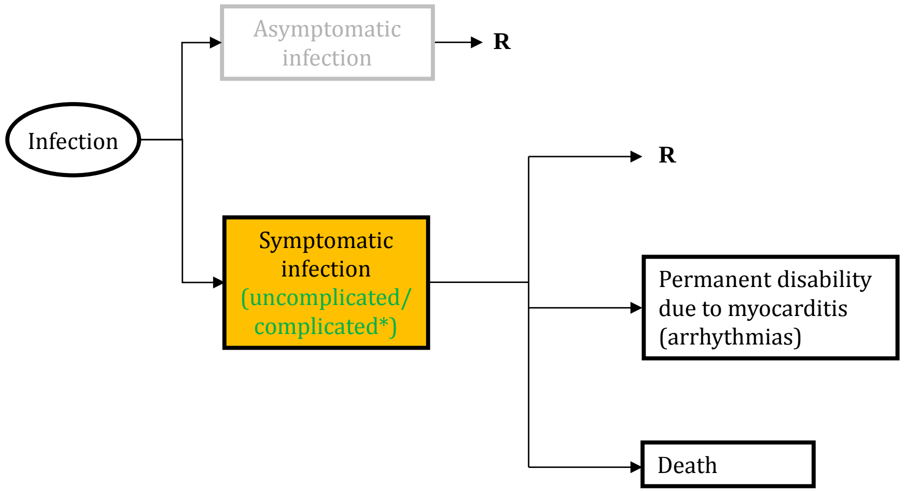

Note. This outcome tree depicts the respiratory variant of diphtheria
* Complicated health states include myocarditis, polyneuropathies/nerve palsies, and systemic toxicity

Health outcome (health state)	Distribution of health states in health outcome	Risk to develop that health outcome	Disability weight (w)	Duration in years
Acute respiratory diphtheria			0.057[a] [24]	0.01918<br>Estimated from [25]<br>(10 days to 3 months)
	10–25% myocarditis [23]		0.057[a] [24]	0.04247 [26] (10 days to 3 months)
	20% polyneuropathies/ nerve palsies [23]		0.057[a] [24]	0.1781 [23, 26]
Permanent disability due to myocarditis (arrhythmias)		0.25% [26]	0.295[b] [24]	RLE [26]
Death following diphtheria		6% [c]		

RLE=remaining life expectancy
[a] Syndromic weight acute episode: 92% moderate (0.051), 8% severe (0.125), [b] Disability weight for cardiac conduction disorders & dysrhythmias, [c] in the original BCoDE-model the following mortality was used: 2.1–4.2% following acute illness, 2.5–5% following myocarditis, and 0.25–0.5% following polyneuropathies/nerve palsies [27], we decided to use age-specific mortality figures in 1952 which were considerably higher (173 registered deaths/2,805 cases=6% mortality on average) (1950: 196 registered deaths/2,985 cases).

Table A4.2 Outcome tree and disease specific parameters: hepatitis B infection

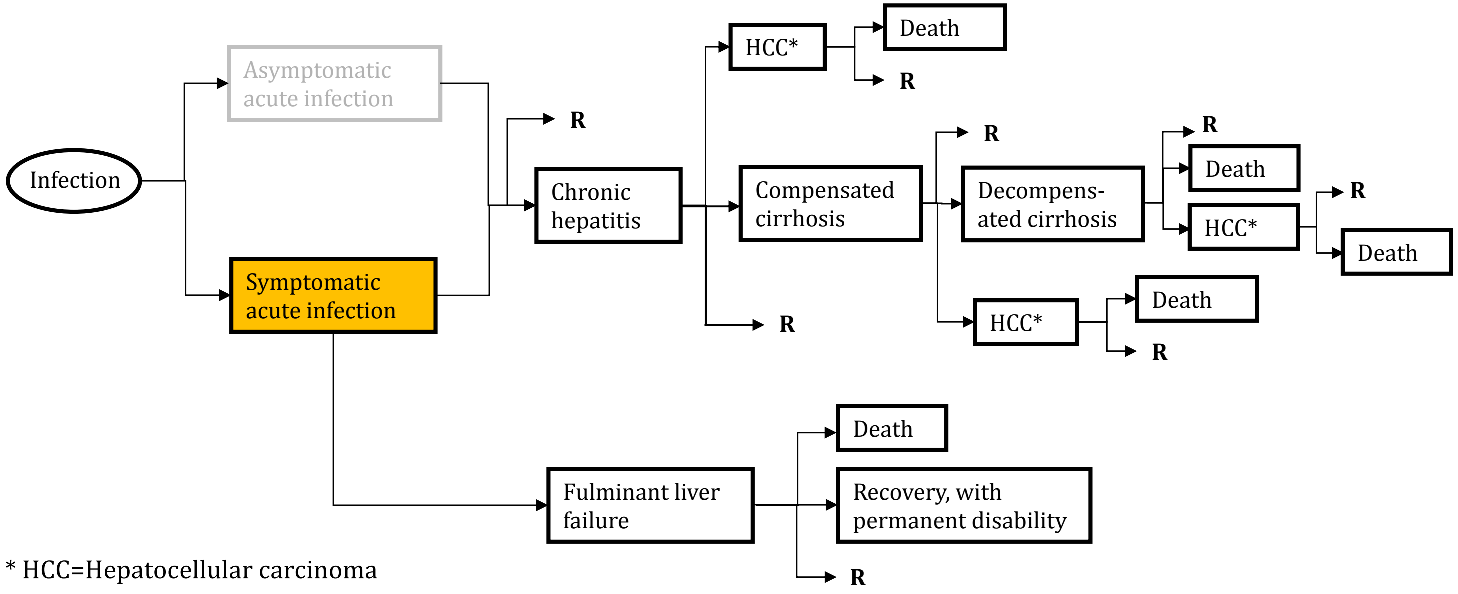

* HCC=Hepatocellular carcinoma

Health outcome (health state)	Distribution of health states in health outcome	Risk to develop that health outcome	Disability weight (w)	Duration in years
Symptomatic infection			0.051[a] [24]	0.17 [28]
Chronic hepatitis		Age-dependent [29]	0.07[b] [24]	37.74 (4.5 active replication) [30]
Fulminant liver failure		0.5–1% [31, 32]	0.515[c] [24]	0.0918 [33]
Compensated cirrhosis		2.1%/year [34]	0.07[b] [24]	Age and sex-dependent [28]
Decompensated cirrhosis		6%/year (5–7%/year) [35]	0.163 [24]	1.429, calculated as 1/prop. leaving compart
HCC, *following*<br>-Chronic hepatitis<br>-Compensated cirrhosis<br>-Decompensated cirrhosis		 5.5%/year (0.1–1%/year) [34]<br>3%/year [35]<br>3%/year [35]	0.265[d] [24]	Age and sex-dependent [28]
Death, *following*:<br>-Fulminant liver failure<br>-Decompensated cirrhosis<br>-HCC		 26.7% (20–33.3%) [36, 37]<br>67%/year (57–77%/year) [35]<br>49%/year [35]		

[a] Acute episode, moderate, [b] generic uncomplicated disease: worry and daily medication, [c] terminal phase, with medication, [d] cancer, diagnosis and primary therapy.

Table A4.3 Outcome tree and disease specific parameters: invasive *Haemophilus influenzae* disease

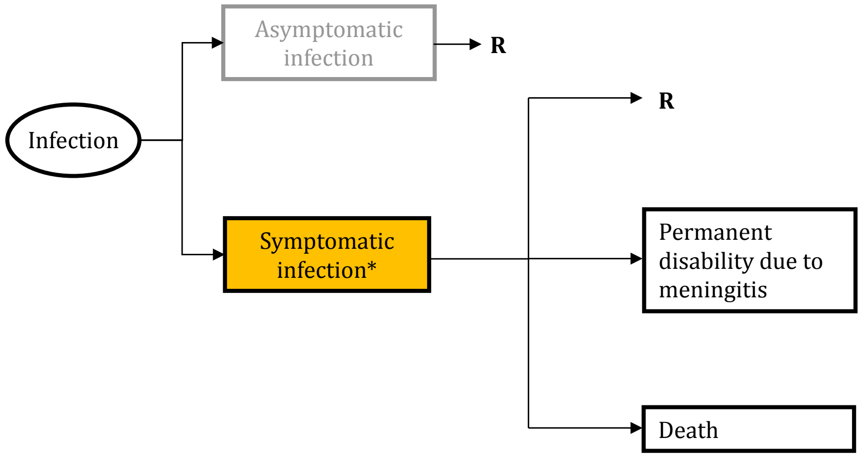

* Health states include bone/joint infections and meningitis; further complications can include the non-invasive forms epiglottitis and pneumonia (although no burden is computed for these).

Health outcome (health state)	Distribution of health states in health outcome	Risk to develop that health outcome	Disability weight (w)	Duration in years
Invasive *H. influenzae* disease	3% bone and joint infections [38, 39]<br><br>55% (50–60%) meningitis [40, 41]<br><br>*Remaining cases are uncomplicated cases*		0.655[a] [24]	0.00274, 1 day (estimated)
Permanent disability due to meningitis: hearing loss or mental retardation		15–30% [42-44]	0.41[b] [24]	RLE [42, 45]
Death following acute illness		5–10%<br>Estimated, based on [46-51]		

RLE=remaining life expectancy
[a] ICU admission, [b] encephalopathy, moderate.

Table A4.4 Outcome tree and disease specific parameters: invasive meningococcal disease (C/W/B)

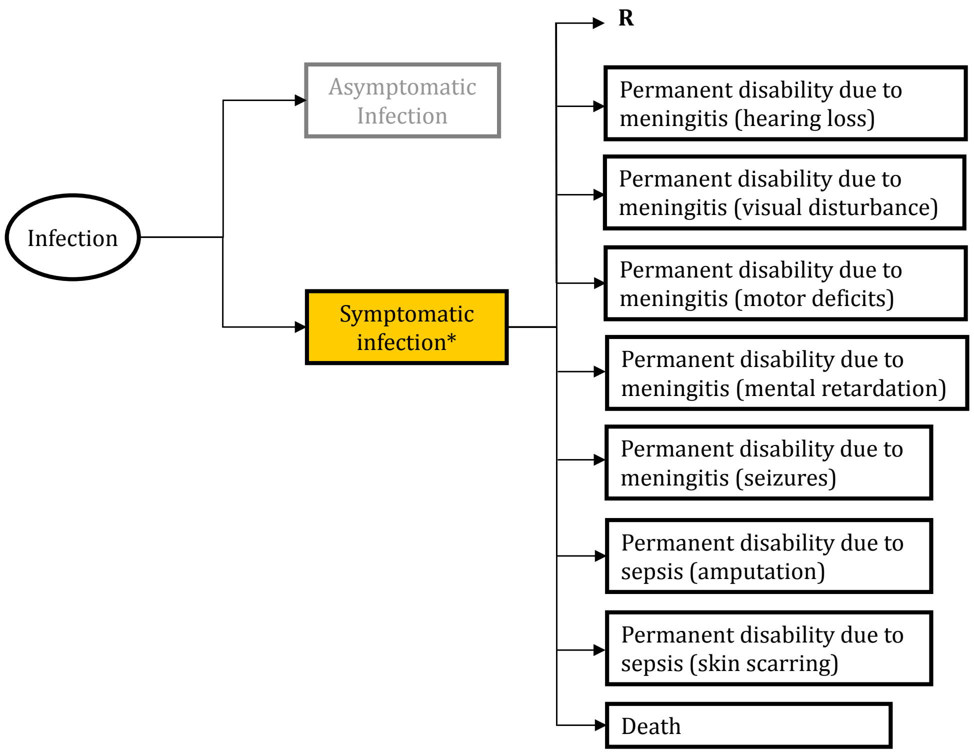

* Health states include meningitis and sepsis; further complications can include the non-invasive form pneumonia (although no burden is computed for this).

Health outcome (health state)	Distribution of health states in health outcome	Risk to develop that health outcome	Disability weight (w)	Duration in years
Invasive meningococcal disease	47.3% meningitis [49]<br>12.5% (5-20%) sepsis [49]<br><br>*Remaining cases are uncomplicated cases*		0.655[a] [24]	0.0192 [52]
Permanent disability due to meningitis: hearing loss		2.1%[a] [53, 54]	0.037[b] [24]	RLE [26, 49]
Permanent disability due to meningitis: visual disturbances		2.1%[a] [53, 54]	0.034[c] [24]	RLE [26, 49]
Permanent disability due to meningitis: motor deficits		0.8%[a] [53, 54]	0.053[d] [24]	RLE [26, 49]
Permanent disability due to meningitis: mental retardation		0.4%[a] [54]	0.123[e] [24]	RLE [26, 49]
Permanent disability due to meningitis: seizure disorders		0.5%[a] [54]	0.488[f] [24]	RLE [26, 49]
Permanent disability due to sepis: amputation		8%[b] [55]	0.041[g] [24]	RLE [55]
Permanent disability due to sepsis: skin scarring		48%[b] [55]	0.067[h] [24]	RLE [55]
Death following acute illness		9–12% [26, 49]		

RLE=remaining life expectancy

[a] ICU admission, [b] hearing loss, moderate, [c] distance vision, moderate impairment, [d] motor impairment, moderate, [e] intellectual disability, moderate, [f] epilepsy, >= once-a-month seizures, [g] amputation of one lower limb, [h] disfigurement, level 2.

Table A4.5 Outcome tree and disease specific parameters: invasive pneumococcal disease

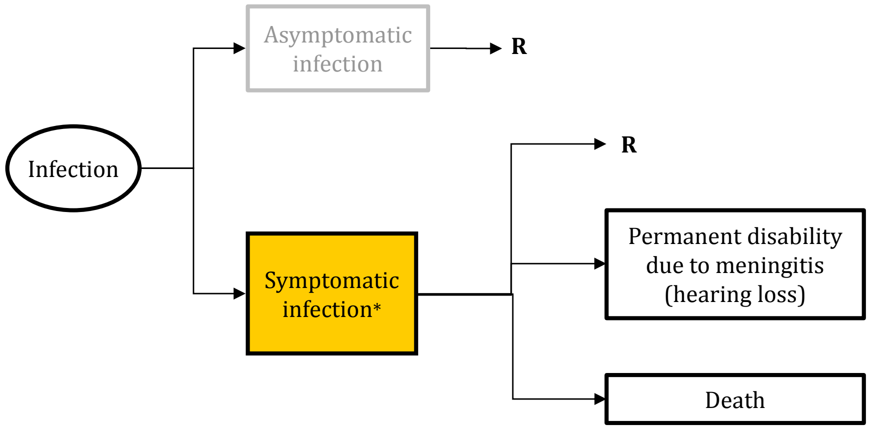

* Health states include meningitis, bacteraemic pneumonia, and bacteraemia without a focus; further complications can include bacteraemia with a focus (endocarditis, osteomyelitis, and septic arthritis, but these are rare), and also the non-invasive forms pneumonia and otitis media. No burden is calculated for the non-invasive complications.

**Health outcome (health state)**	**Distribution of health states in health outcome**	**Risk to develop that health outcome**	**Disability weight (w)**	**Duration in years**
Invasive pneumococcal disease	75% bacteraemia without a focus/bacteraemic pneumonia [56]<br><br>4.5% meningitis [57]<br><br>*Remaining cases are uncomplicated cases*		0.655[a] [24]	0.01356 [57] (2 days)
Permanent disability due to meningitis (hearing loss)		21% [58]	0.037[b] [24]	RLE [58]
Death following invasive pneumococcal disease		10–20% [49, 59-61]		

[a] ICU admission, [b] hearing loss, moderate.

Table A4.6 Outcome tree and disease specific parameters: measles

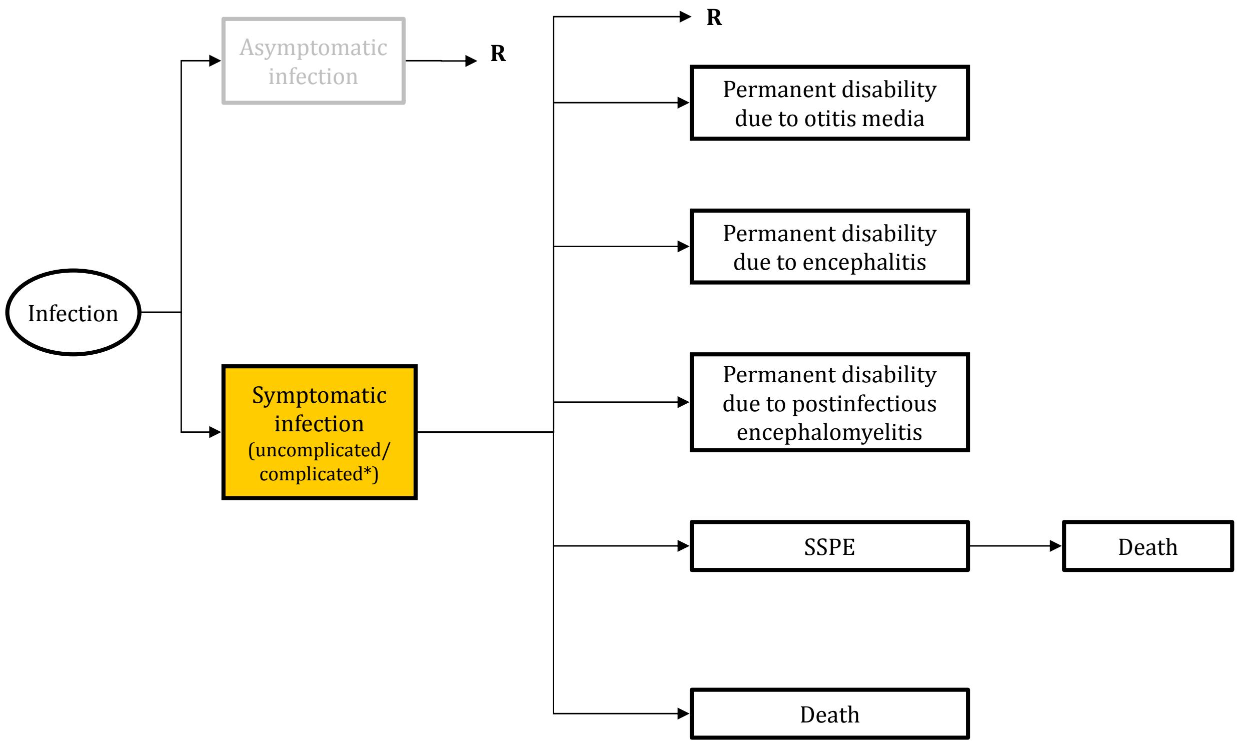

* Complicated health states include pneumonia, otitis media, convulsions, diarrhea, encephalitis, post-infectious encephalomyelitis

**Health outcome (health state)**	**Distribution of health states in health outcome**	**Risk to develop that health outcome**	**Disability weight (w)**	**Duration in years**
Acute measles	5–6% pneumonia [62]<br>8% diarrhea [49]<br>10% otitis media [63]<br>5% convulsions [64]<br>0.1% encephalitis [65-67]<br>0.1–0.3% post-infectious encephalomyelitis (PIE) [68]<br><br>*Remaining cases are uncomplicated cases*		0.071[a] [24]	0.04 [28]
Permanent disability due to otitis media		0.01% [62]	0.037[b] [24]	RLE
Permanent disability due to encephalitis		20–30% [66, 69]	0.185[c] [24]	RLE
Permanent disability due to PIE		25% [62]	0.185[c] [24]	RLE
Subacute sclerosing panencephalitis (SSPE)		0.0081% (<1 year)<br>0.0011% (1-4 years)<br>0.001% (> 5 years) [66]	Pert (0.088, 0.276, 0.543)[d] [24]	2.0 [70]
Death following acute measles		0.007%[e]		

RLE=remaining life expectancy

[a] Syndromic weight acute episode: 73% moderate (0.051), 27% severe (0.125), [b] hearing loss, moderate, [c] motor plus cognitive impairments, moderate, [d] SSPE, phase 1-3,
[e] in the original BCoDE-model the following mortality was used: 100% following subacute sclerosing panencephalitis (SSPE), and 0.10–0.61% [49] following acute measles (based on combined health states), we decided to use age-specific mortality figures in 1976 - assuming one additional SSPE death in the future at 9 years of age [71] - which were considerably lower (1 registered measles death + 1 SSPE death /28,601 cases=0.007% mortality on average).

Table A4.7 Outcome tree and disease specific parameters: mumps

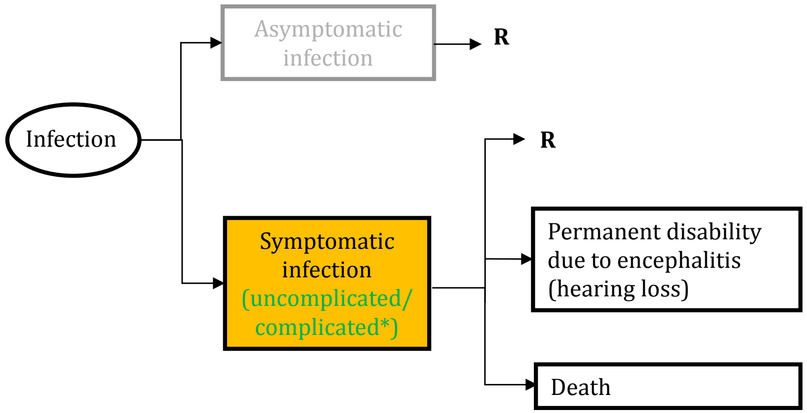

* Complicated health states include orchitis (males only), oophoritis (females only), meningitis, pancreatitis, and encephalitis.

Health outcome (health state)	Distribution of health states in health outcome	Risk to develop that health outcome	Disability weight (w)	Duration in years
Acute mumps	15–30% orchitis [72]<br>5% oophoritis [49]<br>1–10% meningitis [72]<br>0.005–0.1% encephalitis [26, 73]<br>4% pancreatitis [74]<br><br>*Remaining cases are uncomplicated cases*	*(males ≥15 years only)*<br>*(females ≥10 years only)*	0.098[a] [24]	0.03836 [28]
Permanent disability due to encephalitis (hearing loss)		0.005% [72]	0.037[b] [24]	RLE [72]
Death following encephalitis		1.5% [72]		

RLE=remaining life expectancy
[a] syndromic weight acute episode: 36% moderate, 64% severe, [b] hearing loss, moderate.

Table A4.8 Outcome tree and disease specific parameters: pertussis

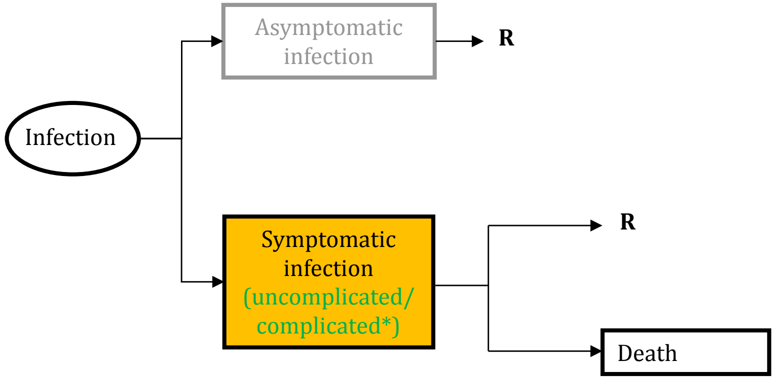

* Complicated health states include pneumonia, otitis media, encephalopathy, and seizures.

Health outcome (health state)	Distribution of health states in health outcome	Risk to develop that health outcome	Disability weight (w)	Duration
Acute pertussis	5.2% pneumonia[a] [49]<br>4% otitis media[a] [75]<br><br>0.1% encephalopathy[a] [49]<br>0.8% seizures[a] [49]<br><br>*Remaining cases are uncomplicated cases*		0.072 (0 yr)<br>0.055 (>0 yr)[b] [24]	0.0767<br>(14 days, estimated)
Death following pertussis		0.01%[c]		

[a] Different from BCoDE project: these percentages were divided by the multiplication factor (depending on age) because it is unlikely that these complications occurred among non-notified cases, [b] syndromic weight acute episode: 0 yr=72% moderate (0.051) and 28% severe (0.125), >0 yr=94% moderate and 6% severe, [c] in the original BCoDE-model the following mortality was used: 7.7% following pneumonia (estimated), 50% following encephalopathy (estimated), and 0.45% (0-9 years)/0.10% (10+ years) following other health states [76], we decided to use age-specific mortality figures in 1953 (81 registered deaths; 145 in 1950) due to lack of notification data (estimated number of cases: 628,486; 1,124,442 in 1950)).

Table A4.9 Outcome tree and disease specific parameters: poliomyelitis

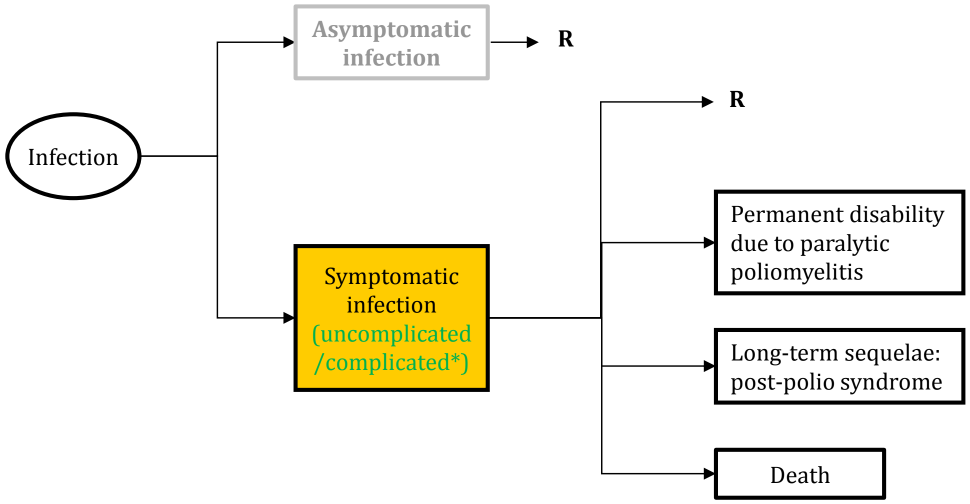

* Complicated health states include non-paralytic poliomyelitis and paralytic poliomyelitis.

Health outcome (health state)	Distribution of health states in health outcome	Risk to develop that health outcome	Disability weight (w)	Duration in years
Poliomyelitis	0.9% other [6]<br>18.3% non-paralytic poliomyelitis [6]<br>80.9%[f] paralytic poliomyelitis [6]		0.007[a] [24]<br>0.051[b] [24]<br>0.125[c] [24]	0.019 [49]<br>0.01643 [49]<br>0.49315 [77]
Permanent disability due to paralytic poliomyelitis		0.5% [78]	0.298[d] [24]	RLE
Postpolio syndrome		25–50% [79]	0.344[e] [24]	RLE
Death following paralytic poliomyelitis		3.4%[g]		

RLE=remaining life expectancy
[a] Acute episode, mild, [b] acute episode, moderate, [c] acute episode, severe, [d] spinal cord lesion below neck (treated), [e] musculoskeletal problem, generalised, moderate,
[f] in the original BCoDE-model paralytic poliomyelitis was assumed to occur in 11.8% of all cases (including uncomplicated cases) based on literature, because we did not correct for underestimation we decided to use age specific figures among reported cases of the outbreak in 1956 in which this percentage was much higher (80.9% on average),
[g] in the original BCoDE-model 5-10% was used, we decided to use the age-specific mortality figures in 1956 which were lower (74 registered deaths/2,206 cases=3.4% mortality on average).

Table A4.10 Outcome tree and disease specific parameters: rotavirus infection

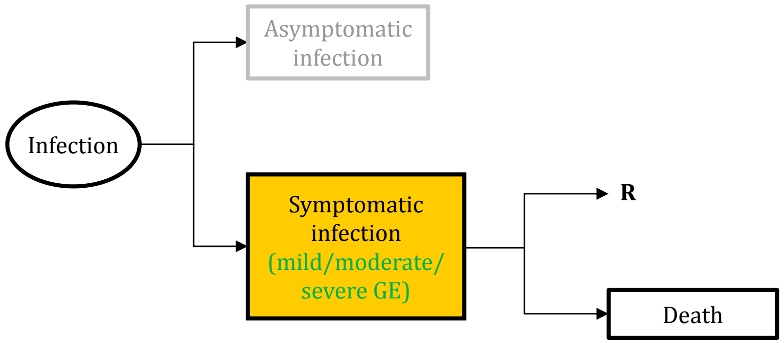

Health outcome (health state)	Distribution of health states in health outcome	Risk to develop that health outcome	Disability weight (w)	Duration in years
Gastroenteritis (GE)<br>- mild (i.e. not visiting a GP)<br>- moderate (i.e. visiting a GP)<br>- severe (i.e. hospitalised)	Modelled, for details see Havelaar et al., 2012 [11].		0.073[a] [24]<br>0.149[b] [24]<br>0.239[c] [24]	0.01 [80]<br>0.02 [80]<br>0.02 [80]
Death following acute illness		Age specific case fatality ratios based on German surveillance data; for details see Havelaar et al. 2012 [11, 81].		

[a] Diarrhea, mild, [b] diarrhea, moderate, [c] diarrhea, severe.

Table A4.11 Outcome tree and disease specific parameters: rubella

Rubella (non-congenital)

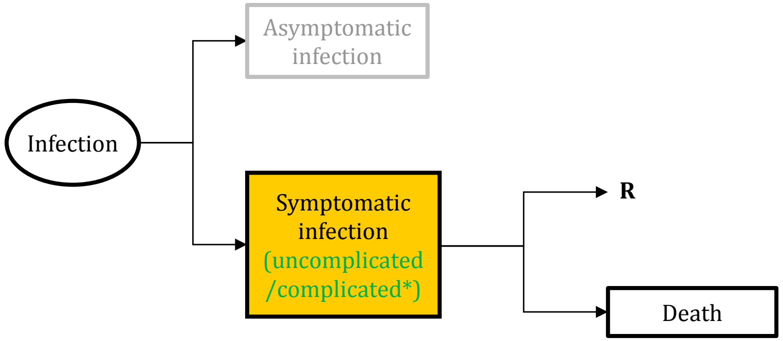

* Complicated health states include arthritis/arthralgia, thrombocytopenic purpura, and encephalitis.

Rubella (CRS)

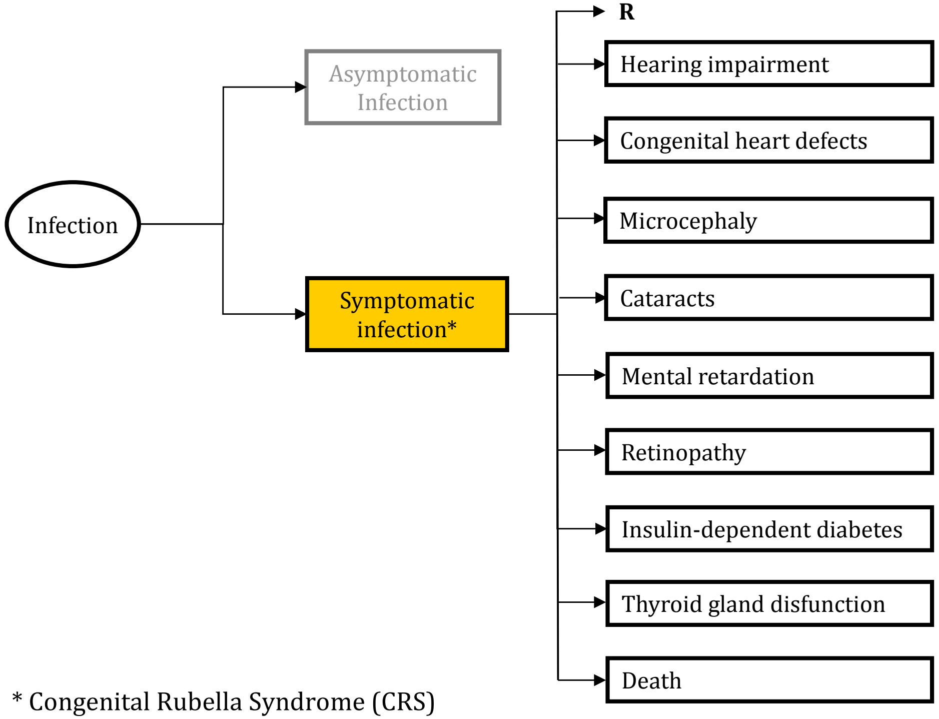

* Congenital Rubella Syndrome (CRS)

Health outcome (health state)	Distribution of health states in health outcome	Risk to develop that health outcome	Disability weight (w)	Duration in years
Non-congenital rubella				
Acute rubella			0.172[a] [24]	0.008219 [49] (3 days)
	Arthritis/arthralgia	30-70% [26, 49, 82] (females only)		
	Thrombocytopenic purpura	0.03% [49, 83]		
	Encephalitis	0.01-0.02% [26, 49]		
	Remaining cases are uncomplicated cases			
Death following thrombocytopenic purpura		4% [83]		
Death following encephalitis		20-50% [26, 83-86]		
Congenital rubella syndrome (CRS; infants)				
Hearing impairment		60% [87]	0.037[b] [24]	RLE
Heart disease		45% [87]	0.07[c] [24]	RLE
Microcephaly		27% [87]	0.123[d] [24]	RLE
Cataract		16-25% [88]	0.034[e] [24]	RLE
Mental retardation		13-25% [87, 89]	0.123[d] [24]	RLE
Retinopathy		5% [87]	0.034[e] [24]	RLE
Insulin-dependent diabetes		20-40% (> 35 years) [26, 90]	0.07[f] [24]	RLE
Thyroid disease		5% (10-19 years) [90]	0.07[f] [24]	RLE
Death due to acute illness		10% [87]		

RLE=remaining life expectancy

[a] Syndromic weight acute episode: 50% arthritis (0.344), 0.03% thrombocytopenic purpura (0.167), 0.015% encephalitis (0.41), remaining mild (0.007), [b] hearing loss, moderate, [c] heart failure, moderate, [d] intellectual disability, moderate, [e] distance vision impairment, moderate, [f] generic uncomplicated disease: worry and daily medication.

Table A4.12 Outcome tree and disease specific parameters: tetanus

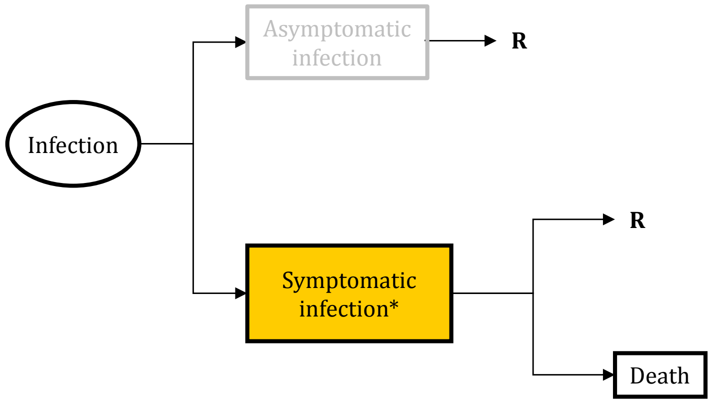

*The health-outcome symptomatic infection is split into three forms (health states) of acute infection: “localised”, “generalised” and “cephalic”

Health outcome (health state)	Distribution of health states in health outcome	Risk to develop that health outcome	Disability weight (w)	Duration in years
Acute tetanus	80% generalized [91, 92]<br>14% localized [91, 92]<br>6% cephalic [93]		0.421 [24]<br>0.011 [24]<br>0.053 [24]	0.04 [28, 91]<br>0.05 [91]<br>0.05[a] [91]
Death following tetanus		100%[b]		

[a] Assumed same as for localised tetanus, [b] in the original BCoDE-model the following mortality was used: 11% following generalised tetanus, 1% following localised tetanus [91], and 15-30% following cephalic tetanus [92], for tetanus we assumed each reported case in 1953 (and 1950) (before availability of mechanical ventilation/intensive care) died of the disease rather than using mortality data which may be unreliable for tetanus in that period [personal communication].

Table A4.13 Outcome tree and disease specific parameters: varicella / herpes zoster

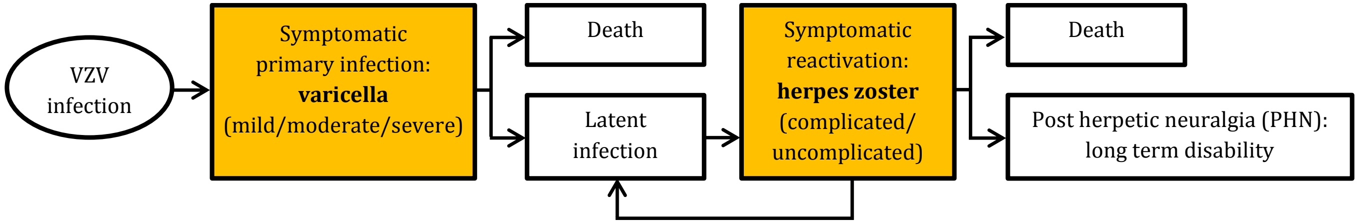

Disease models for varicella and herpes zoster, which are not part of the BCoDE toolkit, were created in the Toolkit by modifying an existing model of another disease using the parameters in the Table below; the relevant files to run the calculation using the Toolkit can be obtained from the corresponding author upon request.

Health outcome (health state)	Distribution of health states in health outcome	Risk to develop that health outcome	Disability weight (w)	Duration in years
Acute varicella	**-Severe varicella** (N=251)<br>Hospitalised cases (based on mean annual number of hospitalisations in 2000–2014 [94])		0.125 [24]	0.038 years (14 days) [96]
	**-Moderate varicella**<br>(N=45 x 1,000)<br>Cases with GP consultation (based on estimated number of GP consultations in 2015 among people <50 years of age* [14])	21.4% with complications [95]<br>78.6% without complications [95]	0.051 [24]<br>0.007 [24]	0.038 years (14 days) [96]<br>0.038 years (14 days) [96]
	Remaining cases are uncomplicated<br>**-Mild varicella**<br>(based on estimated total number of varicella cases according to modelling of VZV seroprevalence data [13] minus the severe and moderate varicella cases)		0.007 [24]	0.038 years (14 days) [96]
Death following severe varicella		Risk was based on mean annual number of deaths by age in 2000–2016 (N=2.5) [97]		

<table>
<tr><th>Health outcome
(health state)</th><th>Distribution of health states in health outcome</th><th colspan="4">Risk to develop that health outcome</th><th>Disability weight (w)</th><th>Duration
in years</th></tr>
<tr><td>Acute herpes zoster</td><td>Complicated herpes zoster
-Meningitis
All ages: 0.1% [98]
-Encephalitis
All ages: 0.1% (estimated)

Remaining cases are uncomplicated</td><td colspan="4"></td><td>0.061 [99]</td><td>0.066 years (24 days) [100]</td></tr>
<tr><td>Death following meningitis</td><td></td><td colspan="4">All ages: 15% (estimated, based on encephalitis)</td><td></td><td></td></tr>
<tr><td>Death following encephalitis</td><td></td><td colspan="4">All ages: 15% (3 out of 20 patients) [101]</td><td></td><td></td></tr>
<tr><td rowspan="8">Post-herpetic neuralgia (PHN) after acute herpes zoster</td><td rowspan="8"></td><td rowspan="2">Age</td><td colspan="3">Pain after:</td><td rowspan="8">0.068 [96]</td><td rowspan="8">Short term pain:
0.16 years
(2 months, estimated)

Long term pain:
2.78 (1.94-4.24) years (1013 (709-1549) days) [102]</td></tr>
<tr><td>1 month [103]</td><td>2 months **</td><td>3 months [103]</td></tr>
<tr><td><45 year</td><td>0.9% (0.2– 2.7%)</td><td>0.3%</td><td>0.3% (0.01– 1.7%)</td></tr>
<tr><td>45-54 year</td><td>3.9% (1.3– 9.0%)</td><td>1.55%</td><td>0.8% (0.02– 4.3%)</td></tr>
<tr><td>55-64 year</td><td>6.5% (3.0–11.9%)</td><td>1.8%</td><td>2.9% (0.8– 7.2%)</td></tr>
<tr><td>65-74 year</td><td>10.7% (5.2–16.3%)</td><td>3.7%</td><td>3.3% (0.9– 8.3%)</td></tr>
<tr><td>≥75 year</td><td>18.0% (11.5–24.6%)</td><td>4.5%</td><td>9.0% (4.8–15.2%)</td></tr>
<tr><td colspan="4">Long term pain=PHN prevalence at 3 months
Short term pain=PHN prevalence at 2 months**</td></tr>
</table>

* Varicella cases in people aged 50 years or older are only sporadically reported by GPs and are therefore not included in the moderate health state.

** To calculate the prevalence of PHN at 2 months, the average between the prevalence at 1 and 3 months was calculated and patients with pain longer than 3 months were subtracted (to avoid double counting).

References

1. WHO methods and data sources for global burden of disease estimates 2000-2011. Geneva: World Health Organization; 2013 (Global Health Estimates Technical Paper WHO/HIS/HSI/GHE/2013.4). http://www.who.int/healthinfo/statistics/GlobalDALYmethods_2000_2011.pdf.
2. Statistics Netherlands. Life expectancy; sex, age (per year and period of 5 years). Voorburg: CBS; 2017; Available from: http://statline.cbs.nl/Statweb/publication/?DM=SLNL&PA=37360ned&D1=3&D2=0&D3=a&D4=20-21,25,45,49,61,68,79,83,89,l&HDR=G1,T,G3&STB=G2&VW=T.
3. Verslag over 1952 van de Geneeskundig Hoofdinspecteur van Volksgezondheid. [Annual report Health Care Inspectorate 1952].1956.
4. van den Hof S, Conyn-van Spaendonck MAE, de Melker HE, Geubbels ELPE, Suijkerbuijk AWM, Talsma E, et al. The effects of vaccination, the incidence of the target diseases. Bilthoven: National Institute for Public Health and the Environment (RIVM); 1998 (RIVM report 213676008).
5. Verslag over 1953 van de Geneeskundig Hoofdinspecteur van Volksgezondheid. [Annual report Health Care Inspectorate 1953].1957.
6. Verslag over 1956 van de Geneeskundig Hoofdinspecteur van Volksgezondheid. [Annual report Health Care Inspectorate 1956].1959.
7. Jaarverslag 1975 van de Geneeskundig Hoofdinspecteur van Volksgezondheid. [Annual report Health Care Inspectorate 1975]. Den Haag: Ministerie van Volksgezondheid en Milieuhygiëne; 1976.
8. Jaarverslag 1977 van de Geneeskundig Hoofdinspecteur van Volksgezondheid. [Annual report Health Care Inspectorate 1977]. Den Haag: Ministerie van Volksgezondheid en Milieuhygiëne; 1978.
9. Havelaar AH, de Wit MA, van Koningsveld R, van Kempen E. Health burden in the Netherlands due to infection with thermophilic Campylobacter spp. Epidemiol Infect. 2000;125(3):505-22.
10. Havelaar AH, Van Duynhoven YT, Nauta MJ, Bouwknegt M, Heuvelink AE, De Wit GA, et al. Disease burden in The Netherlands due to infections with Shiga toxin-producing Escherichia coli O157. Epidemiol Infect. 2004;132(3):467-84.
11. Havelaar AH, Haagsma JA, Mangen MJ, Kemmeren JM, Verhoef LP, Vijgen SM, et al. Disease burden of foodborne pathogens in the Netherlands, 2009. Int J Food Microbiol. 2012;156(3):231-8.
12. van Lier A, Smits G, Mollema L, Waaijenborg S, Berbers G, van der Klis F, et al. Varicella zoster virus infection occurs at a relatively young age in The Netherlands. Vaccine. 2013;31(44):5127-33.
13. van Lier A, Lugner A, Opstelten W, Jochemsen P, Wallinga J, Schellevis F, et al. Distribution of Health Effects and Cost-effectiveness of Varicella Vaccination are Shaped by the Impact on Herpes Zoster. EBioMedicine. 2015;2(10):1494-9.
14. Schurink-van 't Klooster TM, de Melker HE. The National Immunisation Programme in the Netherlands – Surveillance and developments in 2016–2017. Bilthoven: National Institute for Public Health and the Environment (RIVM); 2017 (RIVM report 2017-0143).
15. Mangen MJ, Friesema IHM, Haagsma JA, Van Pelt W. Disease burden of food-related pathogens in the Netherlands, 2016. Bilthoven: National Institute for Public Health and the Environment (RIVM); 2017 (RIVM Letter report 2017-0097).
16. Ramsay M, Gay N, Balogun K, Collins M. Control of hepatitis B in the United Kingdom. Vaccine. 1998;16 Suppl:S52-5.
17. van Isterdael CE, van Essen GA, Kuyvenhoven MM, Hoes AW, Stalman WA, de Wit NJ. Measles incidence estimations based on the notification by general practitioners were suboptimal. J Clin Epidemiol. 2004;57(6):633-7.
18. Greenland K, Whelan J, Fanoy E, Borgert M, Hulshof K, Yap KB, et al. Mumps outbreak among vaccinated university students associated with a large party, the Netherlands, 2010. Vaccine. 2012;30(31):4676-80.
19. Ladbury G, Ostendorf S, Waegemaekers T, van Binnendijk R, Boot H, Hahne S. Smoking and older age associated with mumps in an outbreak in a group of highly-vaccinated individuals attending a youth club party, the Netherlands, 2012. Euro Surveill. 2014;19(16):20776.
20. McDonald SA, Teunis P, van der Maas N, de Greeff S, de Melker H, Kretzschmar ME. An evidence synthesis approach to estimating the incidence of symptomatic pertussis infection in the Netherlands, 2005-2011. BMC Infect Dis. 2015;15:588.
21. Christiansen AH, Andersen PH. [Incidence of tetanus in Denmark, 1983-2000]. Ugeskr Laeger. 2005;167(7):757-9.
22. Opstelten W, van Loon AM, Schuller M, van Wijck AJ, van Essen GA, Moons KG, et al. Clinical diagnosis of herpes zoster in family practice. Ann Fam Med. 2007;5(4):305-9.
23. Hadfield TL, McEvoy P, Polotsky Y, Tzinserling VA, Yakovlev AA. The pathology of diphtheria. J Infect Dis. 2000;181 Suppl 1:S116-20.
24. Haagsma JA, Maertens de Noordhout C, Polinder S, Vos T, Havelaar AH, Cassini A, et al. Assessing disability weights based on the responses of 30,660 people from four European countries. Popul Health Metr. 2015;13:10.

25. Lodha R, Dash NR, Kapil A, Kabra SK. Diphtheria in urban slums in north India. Lancet. 2000;355(9199):204.
26. Mandell GL, Douglas RG, Bennet JE, Dolin R. Mandell, Douglas and Bennett's principles and practice of infectious disease. Philadelphia: Churchill Livingstone; 1999.
27. Rakhmanova AG, Lumio J, Groundstroem K, Valova E, Nosikova E, Tanasijchuk T, et al. Diphtheria outbreak in St. Petersburg: clinical characteristics of 1860 adult patients. Scand J Infect Dis. 1996;28(1):37-40.
28. Murray CJL, Lopez AD. Global health statistics: a compendium of incidence, prevalence and mortality estimates for over 200 conditions (Global burden of disease and injuries series; II). Cambridge: Harvard School of Public Health on behalf of the World Health Organization and the World Bank; 1996.
29. McMahon BJ, Alward WL, Hall DB, Heyward WL, Bender TR, Francis DP, et al. Acute hepatitis B virus infection: relation of age to the clinical expression of disease and subsequent development of the carrier state. J Infect Dis. 1985;151(4):599-603.
30. Virology online. http://virology-online.com/viruses/HepatitisB.htm.
31. Pappas SC. Fulminant viral hepatitis. Gastroenterol Clin North Am. 1995;24(1):161-73.
32. Hoofnagle JH, Doo E, Liang TJ, Fleischer R, Lok AS. Management of hepatitis B: summary of a clinical research workshop. Hepatology. 2007;45(4):1056-75.
33. Kim WR, Brown RS, Jr., Terrault NA, El-Serag H. Burden of liver disease in the United States: summary of a workshop. Hepatology. 2002;36(1):227-42.
34. Chu CM. Natural history of chronic hepatitis B virus infection in adults with emphasis on the occurrence of cirrhosis and hepatocellular carcinoma. J Gastroenterol Hepatol. 2000;15 Suppl:E25-30.
35. D'Amico G, Garcia-Tsao G, Pagliaro L. Natural history and prognostic indicators of survival in cirrhosis: a systematic review of 118 studies. J Hepatol. 2006;44(1):217-31.
36. Bernuau J, Rueff B, Benhamou JP. Fulminant and subfulminant liver failure: definitions and causes. Semin Liver Dis. 1986;6(2):97-106.
37. Wai CT, Fontana RJ, Polson J, Hussain M, Shakil AO, Han SH, et al. Clinical outcome and virological characteristics of hepatitis B-related acute liver failure in the United States. J Viral Hepat. 2005;12(2):192-8.
38. DiLiberti JH, Tarlow S. Bone and joint complications of Hemophilus influenzae meningitis. Clin Pediatr (Phila). 1983;22(1):7-10.
39. Otero Reigada MC, Sanchez Precioso S, Perez Tamarit D, Asensi Botet F, Santos Durantez M, Lorente Molto F, et al. [Haemophilus influenzae type b osteoarthritis. A report of 7 cases and a review of the literature]. An Esp Pediatr. 1998;49(6):594-602.
40. Funkhouser A, Steinhoff MC, Ward J. Haemophilus influenzae disease and immunization in developing countries. Rev Infect Dis. 1991;13 Suppl 6:S542-54.
41. Wenger JD, Booy R, Heath PT, Moxon ER. Epidemiological impact of conjugate vaccines on invasive disease caused by hemophilus influenza type B. In: Levine MM, Woodrow GC, Kaper JB, Cobon GS, editors. New generation vaccines. New York: Marcel Dekker, Inc; 1997. p. 489-502.
42. Lindberg J, Rosenhall U, Nylen O, Ringner A. Long-term outcome of Hemophilus influenzae meningitis related to antiobiotic treatment. Pediatrics. 1977;60(1):1-6.
43. Ladhani S, Slack MP, Heath PT, von Gottberg A, Chandra M, Ramsay ME, et al. Invasive Haemophilus influenzae Disease, Europe, 1996-2006. Emerg Infect Dis. 2010;16(3):455-63.
44. Hwang KW. Haemophilus influenza type b (Hib) vaccine and its carrier proteins. Arch Pharm Res. 2010;33(6):793-5.
45. Naghavi M, Abolhassani F, Pourmalek F, Lakeh M, Jafari N, Vaseghi S, et al. The burden of disease and injury in Iran 2003. Popul Health Metr. 2009;7:9.
46. Dworkin MS, Park L, Borchardt SM. The changing epidemiology of invasive Haemophilus influenzae disease, especially in persons > or = 65 years old. Clin Infect Dis. 2007;44(6):810-6.
47. Cochi SL, Broome CV, Hightower AW. Immunization of US children with Hemophilus influenzae type b polysaccharide vaccine. A cost-effectiveness model of strategy assessment. JAMA. 1985;253(4):521-9.
48. Peltola H, Kilpi T, Anttila M. Rapid disappearance of Haemophilus influenzae type b meningitis after routine childhood immunisation with conjugate vaccines. Lancet. 1992;340(8819):592-4.
49. Centers for Disease Control and Prevention (CDC): Epidemiology and Prevention of Vaccine-Preventable Diseases. Atkinson W, Wolfe S, Hamborsky J, McIntyre L, editors. Washington DC: Public Health Foundation; 2009.
50. Rathore MH, Mirza A. Haemophilus Influenzae Infection. 2010. http://emedicine.medscape.com/article/964317-overview.
51. Thoon KC, Chong CY, Ng WY, Kilgore PE, Nyambat B. Epidemiology of invasive Haemophilus influenzae type b disease in Singapore children, 1994-2003. Vaccine. 2007;25(35):6482-9.
52. Brigham KS, Sandora TJ. Neisseria meningitidis: epidemiology, treatment and prevention in adolescents. Curr Opin Pediatr. 2009;21(4):437-43.
53. Smith DS, Hoffman TA, Chan JL. Meningcococcal infections. [5 September 2011]; Available from: http://emedicine.medscape.com/article/221321.

54. Edmond K, Clark A, Korczak VS, Sanderson C, Griffiths UK, Rudan I. Global and regional risk of disabling sequelae from bacterial meningitis: a systematic review and meta-analysis. Lancet Infect Dis. 2010;10(5):317-28.
55. Buysse CM, Oranje AP, Zuidema E, Hazelzet JA, Hop WC, Diepstraten AF, et al. Long-term skin scarring and orthopaedic sequelae in survivors of meningococcal septic shock. Arch Dis Child. 2009;94(5):381-6.
56. Harper MB, Flaisher GR. Infectious disease emergencies. In: Fleisher GR, Ludwig S, editors. Textbook of Pediatric Emergency Medicine. Philadelphia: Lippincott Williams & Wilkins; 2010.
57. Muller ML. Pneumococcal Bacteremia. 2009. http://emedicine.medscape.com/article/967600-overview.
58. Jit M. The risk of sequelae due to pneumococcal meningitis in high-income countries: a systematic review and meta-analysis. J Infect. 2010;61(2):114-24.
59. Rudan I, Campbell H. The deadly toll of S pneumoniae and H influenzae type b. Lancet. 2009;374(9693):854-6.
60. Lin SH, Lai CC, Tan CK, Liao WH, Hsueh PR. Outcomes of hospitalized patients with bacteraemic and non-bacteraemic community-acquired pneumonia caused by Streptococcus pneumoniae. Epidemiol Infect. 2011;139(9):1307-16.
61. Saldias PF, Viviani GP, Pulgar BD, Valenzuela FF, Paredes ES, Diaz PO. [Prognostic factors and mortality in immunocompetent adult patients hospitalized with community-acquired pneumococcal pneumonia]. Rev Med Chil. 2009;137(12):1545-52.
62. Centers for Disease C. Measles--United States, 1990. MMWR Morb Mortal Wkly Rep. 1991;40(22):369-72.
63. Centers for Disease Control and Prevention (CDC): Complications of measles. [23 August 2011]; Available from: http://www.cdc.gov/measles/about/complications.html.
64. Miller CL. Severity of notified measles. Br Med J. 1978;1(6122):1253.
65. Weissbrich B, Schneider-Schaulies J, ter Meulen V. Measles and its neurological complications. In: Nath A, Berger JA, editors. Clinical Neurovirology. New York: Marcel Dekker; 2003.
66. Beutels P, Van Damme P, Van Casteren V, Gay NJ, De Schrijver K, Meheus A. The difficult quest for data on "vanishing" vaccine-preventable infections in Europe: the case of measles in Flanders (Belgium). Vaccine. 2002;20(29-30):3551-9.
67. Miller HG, Stanton JB, Gibbons JL. Acute disseminated encephalomyelitis and related syndromes. Br Med J. 1957;1(5020):668-72.
68. Perry RT, Halsey NA. The clinical significance of measles: a review. J Infect Dis. 2004;189 Suppl 1:S4-16.
69. Filia A, Brenna A, Pana A, Cavallaro GM, Massari M, Ciofi degli Atti ML. Health burden and economic impact of measles-related hospitalizations in Italy in 2002-2003. BMC Public Health. 2007;7:169.
70. Garg RK. Subacute sclerosing panencephalitis. J Neurol. 2008;255(12):1861-71.
71. Schonberger K, Ludwig MS, Wildner M, Weissbrich B. Epidemiology of subacute sclerosing panencephalitis (SSPE) in Germany from 2003 to 2009: a risk estimation. PLoS One. 2013;8(7):e68909.
72. Hviid A, Rubin S, Muhlemann K. Mumps. Lancet. 2008;371(9616):932-44.
73. Demirci CS, Abuhammour W, Quintana EC, Shahidi H, Wilkes G. Mumps. [12 September 2011]; Available from: http://emedicine.medscape.com/article/966678-overview.
74. Vanlioglu B, Chua TC. Presentation of mumps infection as acute pancreatitis without parotitis. Pancreas. 2011;40(1):167-8.
75. De Serres G, Shadmani R, Duval B, Boulianne N, Dery P, Douville Fradet M, et al. Morbidity of pertussis in adolescents and adults. J Infect Dis. 2000;182(1):174-9.
76. Rothstein E, Edwards K. Health burden of pertussis in adolescents and adults. Pediatr Infect Dis J. 2005;24(5 Suppl):S44-7.
77. Farbu E. Post-polio Syndrome. In: Stone JH, Blouin M, editors. International Encyclopedia of Rehabilitation2013. http://cirrie.buffalo.edu/encyclopedia/en/article/138/.
78. World Health Organization (WHO). Poliomyelitis. [29 August 2011]; Available from: http://www.who.int/topics/poliomyelitis/en/.
79. Jubelt B, Drucket J. Poliomyelitis and the Post-Polio Syndrome. In: Younger DS, editor. Motor Disorders. Philadelphia: Lippincott Williams and Wilkins; 1999. p. 381.
80. Kemmeren JM, Mangen MJJ, Van Duynhoven YTHP, Havelaar AH. Priority setting of foodborne pathogens - Disease burden and costs of selected enteric pathogens. Bilthoven: National Institute for Public Health and the Environment (RIVM); 2006 (RIVM report 330080001).
81. Verhoef L, Koopmans M, W VANP, Duizer E, Haagsma J, Werber D, et al. The estimated disease burden of norovirus in The Netherlands. Epidemiol Infect. 2013;141(3):496-506.
82. Johnson RE, Hall AP. Rubella arthritis; report of cases studied by latex tests. N Engl J Med. 1958;258(15):743-5.
83. White CC, Koplan JP, Orenstein WA. Benefits, risks and costs of immunization for measles, mumps and rubella. Am J Public Health. 1985;75(7):739-44.
84. Gulen F, Cagliyan E, Aydinok Y, Ozen S, Yildiz B. A patient with rubella encephalitis and status epilepticus. Minerva Pediatr. 2008;60(1):141-4.

85. Steen E, Torp KH. Encephalitis and thrombocytopenic purpura after rubella. Arch Dis Child. 1956;31(160):470-3.
86. Sherman FE, Michaels RH, Kenny FM. Acute Encephalopathy (Encephalitis) Complicating Rubella. Report of Cases with Virologic Studies, Cortisol-Production Determinations, and Observations at Autopsy. JAMA. 1965;192:675-81.
87. Reef SE, Plotkin S, Cordero JF, Katz M, Cooper L, Schwartz B, et al. Preparing for elimination of congenital Rubella syndrome (CRS): summary of a workshop on CRS elimination in the United States. Clin Infect Dis. 2000;31(1):85-95.
88. Bloom S, Rguig A, Berraho A, Zniber L, Bouazzaoui N, Zaghloul Z, et al. Congenital rubella syndrome burden in Morocco: a rapid retrospective assessment. Lancet. 2005;365(9454):135-41.
89. Lanzieri TM, Parise MS, Siqueira MM, Fortaleza BM, Segatto TC, Prevots DR. Incidence, clinical features and estimated costs of congenital rubella syndrome after a large rubella outbreak in Recife, Brazil, 1999-2000. Pediatr Infect Dis J. 2004;23(12):1116-22.
90. Duszak RS. Congenital rubella syndrome--major review. Optometry. 2009;80(1):36-43.
91. Centers for Disease Control and Prevention (CDC): Tetanus. In: Atkinson W, Wolfe S, Hamborsky J, editors. Epidemiology and Prevention of Vaccine-Preventable Diseases. Washington DC: Public Health Foundation; 2012. p. 291-300.
92. Hsu SS, Groleau G. Tetanus in the emergency department: a current review. J Emerg Med. 2001;20(4):357-65.
93. Bardenheier B, Prevots DR, Khetsuriani N, Wharton M. Tetanus surveillance--United States, 1995-1997. MMWR CDC Surveill Summ. 1998;47(2):1-13.
94. Dutch Hospital Data. National Medical Register (LMR). Utrecht: Dutch Hospital Data; 2000-2014.
95. van Lier A, van Erp J, Donker GA, van der Maas NA, Sturkenboom MC, de Melker HE. Low varicella-related consultation rate in the Netherlands in primary care data. Vaccine. 2014;32(28):3517-24.
96. Kwong JC, Crowcroft NS, Campitelli MA, Ratnasingham S, Daneman N, Deeks SL, et al. Ontario Burden of Infectious Disease Study (ONBOIDS): An OAHPP/ICES Report. Toronto: Ontario Agency for Health Protection and Promotion, Institute for Clinical Evaluative Sciences; 2010.
97. Statistics Netherlands. Deaths by main primary cause of death, sex and age. Voorburg: CBS; 2000-2016.
98. Hillebrand K, Bricout H, Schulze-Rath R, Schink T, Garbe E. Incidence of herpes zoster and its complications in Germany, 2005-2009. J Infect. 2015;70(2):178-86.
99. Salomon JA, Vos T, Hogan DR, Gagnon M, Naghavi M, Mokdad A, et al. Common values in assessing health outcomes from disease and injury: disability weights measurement study for the Global Burden of Disease Study 2010. Lancet. 2012;380(9859):2129-43.
100. Oxman MN, Levin MJ, Johnson GR, Schmader KE, Straus SE, Gelb LD, et al. A vaccine to prevent herpes zoster and postherpetic neuralgia in older adults. N Engl J Med. 2005;352(22):2271-84.
101. Rottenstreich A, Oz ZK, Oren I. Association between viral load of varicella zoster virus in cerebrospinal fluid and the clinical course of central nervous system infection. Diagn Microbiol Infect Dis. 2014;79(2):174-7.
102. van Hoek AJ, Gay N, Melegaro A, Opstelten W, Edmunds WJ. Estimating the cost-effectiveness of vaccination against herpes zoster in England and Wales. Vaccine. 2009;27(9):1454-67.
103. Opstelten W, Mauritz JW, de Wit NJ, van Wijck AJ, Stalman WA, van Essen GA. Herpes zoster and postherpetic neuralgia: incidence and risk indicators using a general practice research database. Fam Pract. 2002;19(5):471-5.
