# Supplementary material for: Disease burden of varicella versus other vaccine-preventable diseases before introduction of vaccination into the national immunisation programme in the Netherlands
Source: Euro Surveill. 2019 May 2;24(18):1800363. doi: 10.2807/1560-7917.ES.2019.24.18.1800363 (PMC6505181; doi:10.2807/1560-7917.ES.2019.24.18.1800363)
Supplement: Supplement 2 [file 1800363_VanLIER_Supplement2.pdf]

This supplementary material is hosted by *Eurosurveillance* as supporting information alongside the article 'Disease burden of varicella versus other vaccine-preventable diseases before introduction of vaccination into the national immunisation programme in the Netherlands' by van Lier et al. on behalf of the authors who remain responsible for the accuracy and appropriateness of the content. The same standards for ethics, copyright, attributions and permissions as for the article apply. *Eurosurveillance* is not responsible for the maintenance of any links or email addresses provided therein.

Supplement 2 - Additional results

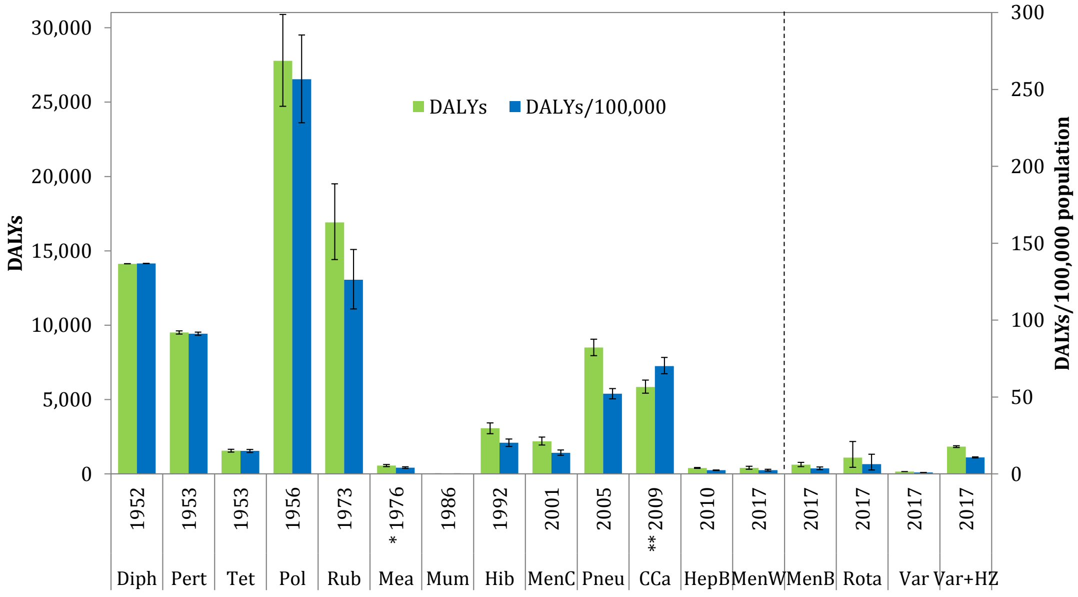

* 1976 because there was no 1975 data, ** Dutch life expectancy in 2014 instead of GBD 2010 life expectancy.
Diph: diphtheria; Pert: pertusis; Tet: tetanus; Pol: poliomyelitis; Rub: rubella; Mea: measles; Mum: mumps; Hib: invasive *Haemophilus influenzae* type b disease; MenC/W/B: invasive meningococcal C/W/B disease; Pneu: invasive pneumococcal disease (PCV10 types); CCa: cervical cancer (human papillomavirus (HPV)-16/18); HepB: hepatitis B; Rota: rotavirus gastroenteritis; Var: varicella; HZ: herpes zoster.

Figure B1 Estimated disease burden of vaccine-preventable diseases in the year before introduction of vaccination into the national immunisation programme, or in 2017, expressed in DALYs (in green) and DALYs per 100,000 population (in blue), Netherlands, 1952–2017

DALY: disability-adjusted life year; CCa per 100,000 *women*; left of vertical dashed line: estimates for the year before inclusion in the national immunisation programme (NIP), right of vertical dashed line: estimates for 2017 for potential NIP candidates; whiskers indicate 95% uncertainty intervals for DALY; see manuscript and Supplement 1 for all assumptions and limitations.

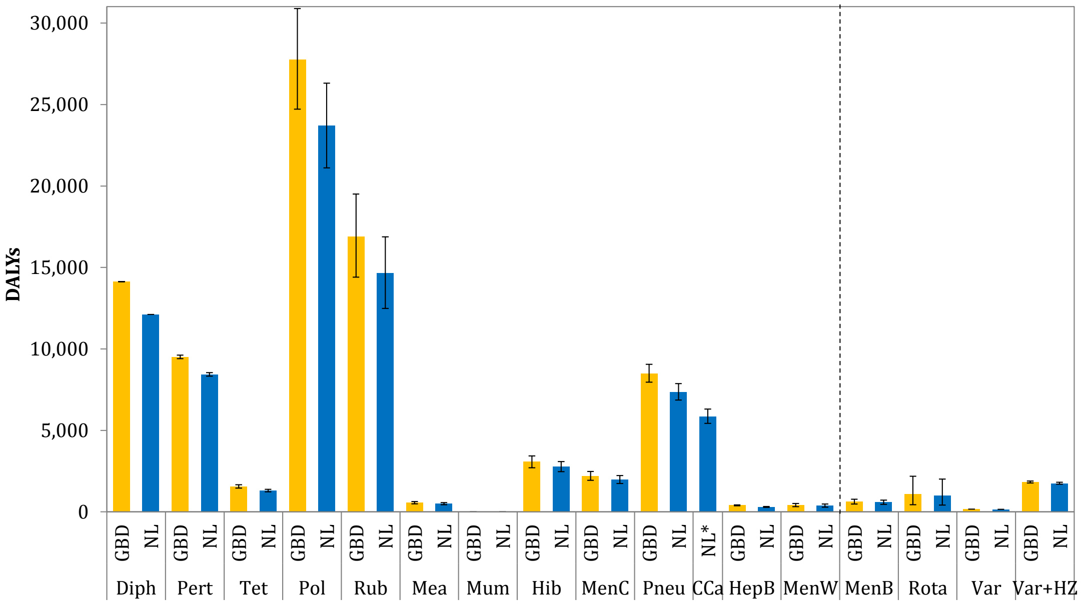

* Dutch life expectancy in 2014 instead of 2008.
Diph: diphtheria; Pert: pertusis; Tet: tetanus; Pol: poliomyelitis; Rub: rubella; Mea: measles; Mum: mumps; Hib: invasive *Haemophilus influenzae* type b disease; MenC/W/B: invasive meningococcal C/W/B disease; Pneu: invasive pneumococcal disease (PCV10 types); CCa: cervical cancer (human papillomavirus (HPV)-16/18); HepB: hepatitis B; Rota: rotavirus gastroenteritis; Var: varicella; HZ: herpes zoster.

Figure B2 Estimated disease burden (expressed in DALYs) of vaccine-preventable diseases in the year before introduction of vaccination into the national immunisation programme, or in 2017, with GBD 2010 life expectancy (baseline analysis, in orange) versus the alternative scenario with year-specific Dutch life expectancy (sensitivity analysis, in blue), Netherlands, 1952–2017

DALY: disability-adjusted life year; left of vertical dashed line: estimates for the year before inclusion in the national immunisation programme (NIP), right of vertical dashed line: estimates for 2017 for potential NIP candidates; whiskers indicate 95% uncertainty intervals for DALY; see manuscript and Supplement 1 for all assumptions and limitations.

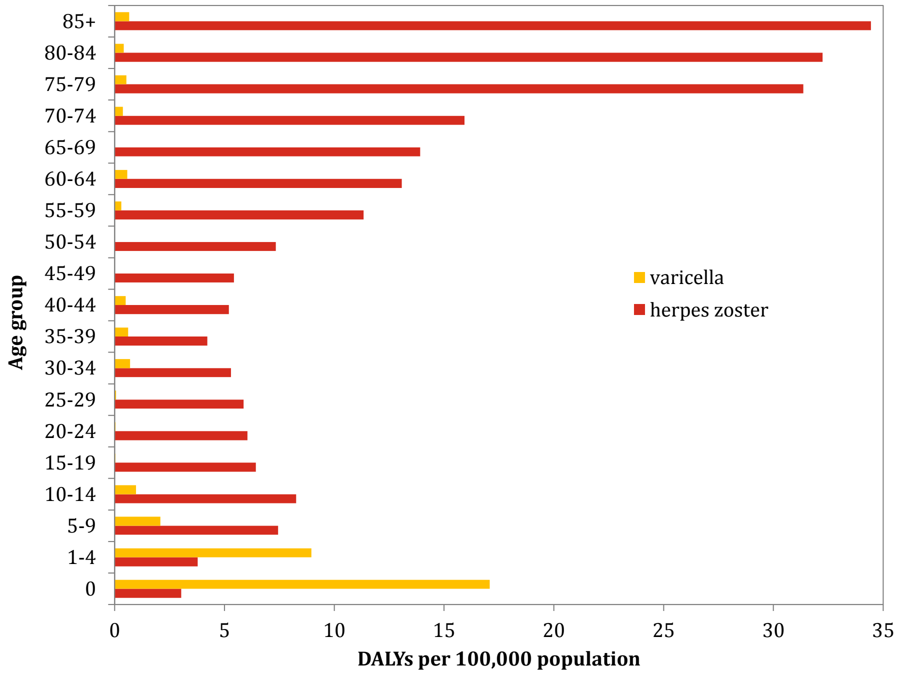

Figure B3 Estimated disease burden (expressed in DALYs) of varicella and herpes zoster per 100,000 population, by age group, Netherlands, 2017

DALY: disability-adjusted life year
